# Influence of Organic Solvents on Enzymatic Asymmetric Carboligations

**DOI:** 10.1002/adsc.201200284

**Published:** 2012-10-04

**Authors:** Tina Gerhards, Ursula Mackfeld, Marco Bocola, Eric von Lieres, Wolfgang Wiechert, Martina Pohl, Dörte Rother

**Affiliations:** a Institute of Bio- and Geosciences, IBG-1: Biotechnology, Forschungszentrum Jülich GmbH52425 Jülich, Germany, Fax: (+49)-2461-613870; phone: (+49)-2461-616772; b Institute of Biotechnology, RWTH Aachen UniversityWorringerweg 1, 52074 Aachen, Germany

**Keywords:** bimolecular carboligation, chemoselectivity, 2-hydroxy ketones, *S*-pocket, stereoselectivity, thiamine diphosphate-dependent enzymes, water-miscible organic solvents

## Abstract

The asymmetric mixed carboligation of aldehydes with thiamine diphosphate (ThDP)-dependent enzymes is an excellent example where activity as well as changes in chemo- and stereoselectivity can be followed sensitively. To elucidate the influence of organic additives in enzymatic carboligation reactions of mixed 2-hydroxy ketones, we present a comparative study of six ThDP-dependent enzymes in 13 water-miscible organic solvents under equivalent reaction conditions. The influence of the additives on the stereoselectivity is most pronounced and follows a general trend. If the enzyme stereoselectivity in aqueous buffer is already >99.9% *ee*, none of the solvents reduces this high selectivity. In contrast, both stereoselectivity and chemoselectivity are strongly influenced if the enzyme is rather unselective in aqueous buffer. For the *S*-selective enzyme with the largest active site, we were able to prove a general correlation of the solvent-excluded volume of the additives with the effect on selectivity changes: the smaller the organic solvent molecule, the higher the impact of this additive. Further, a correlation to log P of the additives on selectivity was detected if two additives have almost the same solvent-excluded volume. The observed results are discussed in terms of structural, biochemical and energetic effects. This work demonstrates the potential of medium engineering as a powerful additional tool for varying enzyme selectivity and thus engineering the product range of biotransformations. It further demonstrates that the use of cosolvents should be carefully planned, as the solvents may compete with the substrate(s) for binding sites in the enzyme active site.

## Introduction

Thiamine diphosphate (ThDP)-dependent enzymes catalyze the reversible cleavage of C–C bonds.^1^ Many of these enzymes catalyze the carboligation of two aldehydes to form pharmaceutically potent 2-hydroxy ketones in an often highly selective manner.^2^ A well-known example is the carboligation of acetaldehyde and benzaldehyde catalyzed by pyruvate decarboxylases in fermenting yeasts yielding (*R*)-1-hydroxy-1-phenylpropan-2-one (PAC), a precursor of ephedrine, with an *ee* >98%.^3^ In recent years, we have created a toolbox of ThDP-dependent enzymes including wild-type and engineered enzymes with varying selectivities to access a broad range of predominantly mixed aromatic and aliphatic chiral 2-hydroxy ketones.2f,^4^ In doing so, different organic cosolvents [e.g., dimethyl sulfoxide (DMSO),^5^ methyl *tert*-butyl ether (MTBE),^6^ 2-methyltetrahydrofuran (MTHF)^7^] were used in order to increase the solubility of the aromatic compounds in the aqueous reaction system. Depending on the enzyme and the reaction studied, the influence of the organic cosolvents ranged between no effect relative to the aqueous system to significant influence on enzyme activity, stability and selectivity. Here, especially DMSO is known to have a stabilizing effect on enzymes^8^ and also on one of the ThDP-dependent enzymes, namely benzaldehyde lyase from *Pseudomonas fluorescens*.^5^,^9^

**Figure 1:**
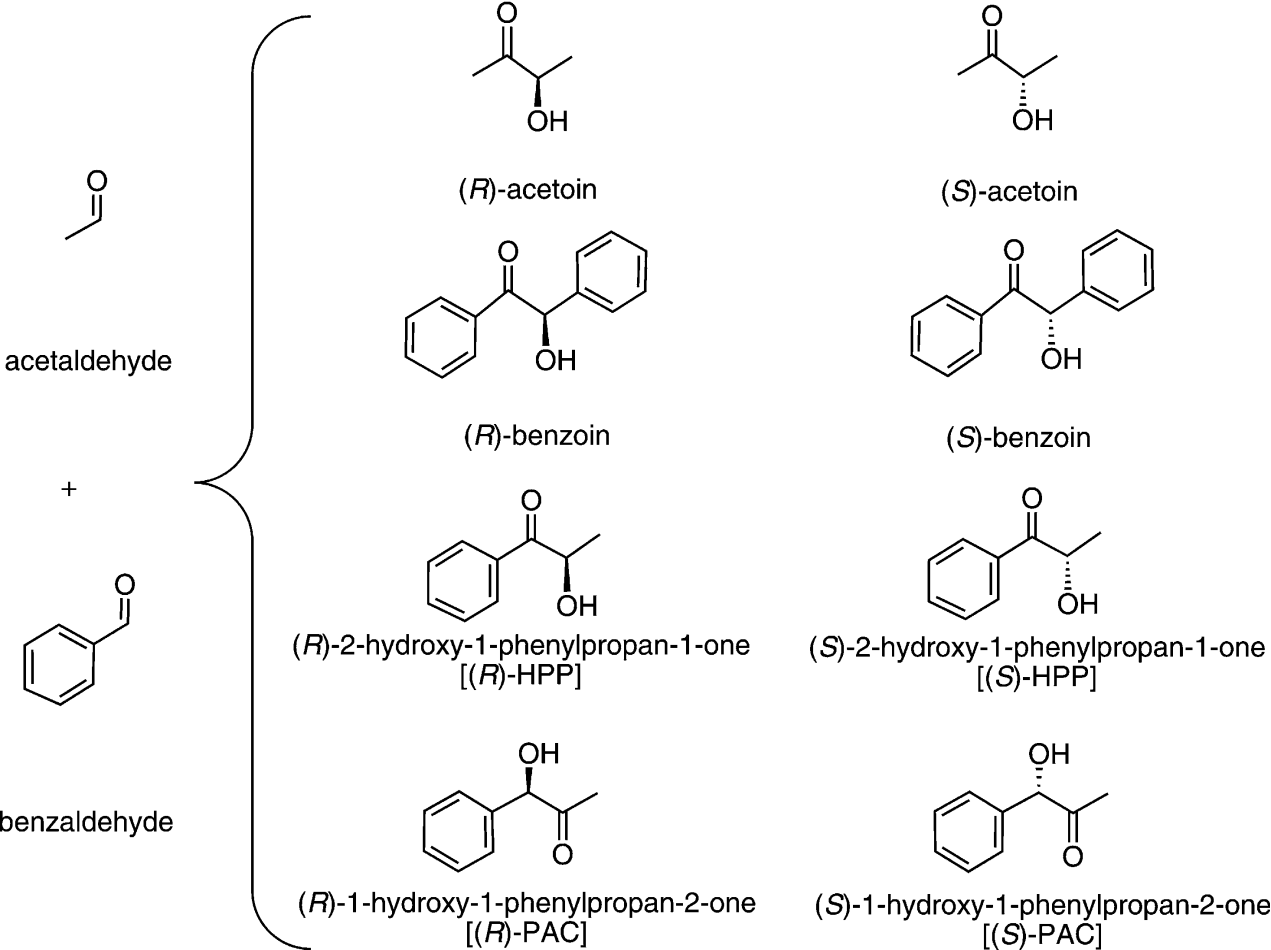
The eight possible different enantiomeric products derived from the carboligation of acetaldehyde and benzaldehyde.

Thus, the goal of the present work was to analyze the influence of organic cosolvents in a more systematic way, using six different enzymes and 13 cosolvents in different concentrations under similar reaction conditions. All six ThDP-dependent enzymes catalyze the carboligation of acetaldehyde and benzaldehyde. This reaction theoretically yields eight different enantiomeric products (Figure 1) each of them in the *R*- or *S*-configuration. However, the product range and the stereochemistry are different for the respective enzymes, which makes them an interesting tool for studying the influence of non-conventional media.

The ThDP-catalyzed carboligation of aldehydes involves a multistep mechanism, which starts with the binding of the donor aldehyde to the ThDP-ylide resulting in an *umpolung* of the carbonyl reactivity yielding a so-called “activated aldehyde” (for details see the Supporting Information, Figure S1). Subsequently, this “activated aldehyde” nucleophilically attacks an acceptor aldehyde. The products acetoin and PAC (Figure 1) result from an acetaldehyde donor and an acetaldehyde or benzaldehyde acceptor, respectively. Benzoin and 2-hydroxy-1-phenylpropan-1-one (HPP) are derived from benzaldehyde as the donor and benzaldehyde or acetaldehyde as the acceptor, respectively. ThDP itself, as the actual catalytic species, and its enzymatic surrounding thus provide a hydrophobic environment in the active site and define the available space for donor and acceptor. As a consequence, many ThDP-dependent enzymes are highly selective for only one or two of the possible products of Figure 1. This donor and acceptor concept directs the chemoselectivity (product range) of ThDP-dependent enzymes.^10^

**Figure 2:**
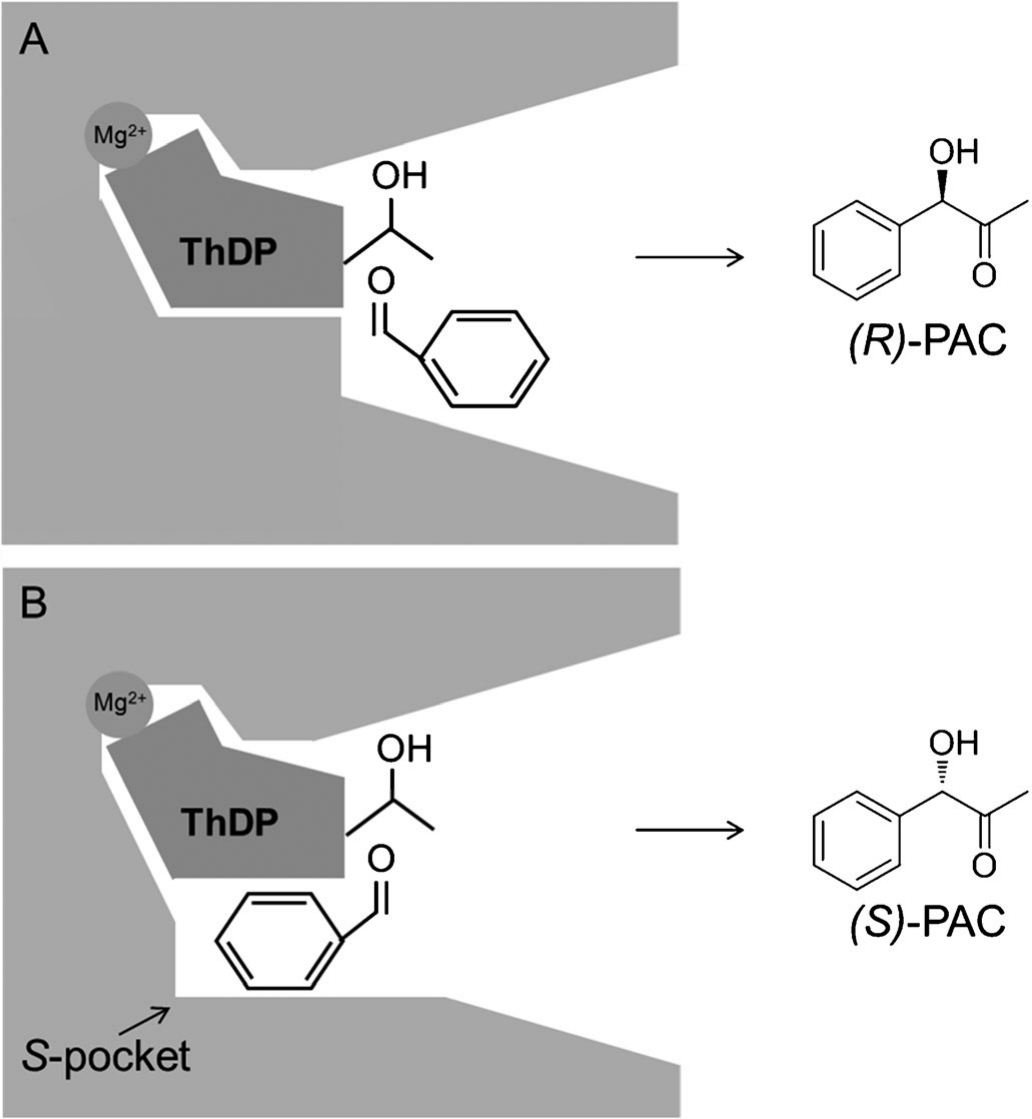
Schematic representation of the active site of ThDP-dependent enzymes showing the situation prior to C–C bond formation. The parallel orientation of donor and acceptor leads to the *R*-product (A), while an antiparallel arrangement in the active site yields the *S*-enantiomer (B). The *S*-configuration is possible due to a steric specialty, the so-called *S*-pocket.^11^

Similar steric principles also control the stereoselectivity. As demonstrated in Figure 2 stereoselectivity can be explained by the relative orientation of the ThDP-bound donor aldehyde to the acceptor aldehyde prior to C–C bond formation.^11^

A special structural element, the so-called *S*-pocket, allows the antiparallel orientation of the two substrate aldehydes in the active site (Figure 2, B), yielding the *S*-product. Due to this principle, most of the currently known ThDP-dependent enzymes are *R*-selective, because an *S*-pocket is missing or blocked by bulky amino acid side chains (Figure 2, A). However, the two available *S*-selective enzymes, benzoylformate decarboxylase from *Pseudomonas putida* (*Pp*BFD2d) and a variant of the pyruvate decarboxylase from *Acetobacter pasteurianus* (*Ap*PDCE469G11d) are selective for the carboligation of (*S*)-HPP and (*S*)-PAC, respectively. Both were included in this study together with four *R*-selective enzymes: branched-chain keto acid decarboxylase from *Lactobacillus lactis* (*Ll*KdcA^12^), benzaldehyde lyase from *Pseudomonas fluorescens* (*Pf*BAL^13^), a variant of *Pp*BFD (*Pp*BFDH281A^10^,^14^) and wild-type *Ap*PDC.11d This set of enzymes with different chemo- and stereoselectivities is well suited for studying effects due to environmental changes.

Up to now, the influence of non-conventional media has mainly been studied with lipases in neat organic solvents.^15^ Some studies proved an influence on the stereoselectivity of lipases^16^ and proteases.^17^ With lipases also stereoinversions were reported, for example, from 39% *ee* (*S*) to 39% *ee* (*R*)15f or even from 60% (*R*) to 67% (*S*).15a Furthermore, several publications describe the effects of mixtures of water and organic solvents on enzymes, for example, the impact of such solvents on the structures of lipases,^18^ alcohol dehydrogenases^19^ and monooxygenases.^20^ Kaul et al. identified some general trends of different water-miscible solvents with respect to the conversion and the stereoselectivity of a nitrilase by correlating the log P value and dielectric constant of a solvent to conversion and initial reaction rates.^21^

Although some studies have been conducted, for example, for the kinetic behaviour of alpha-chymotrypsin in organic solvents,^22^ up to now it was not possible to generalize the influence of organic solvents on enzyme behaviour. Mechanistic explanations are only applicable for very specific systems^23^ and solvent effects in general are by no means completely understood.

First results concerning the application of ThDP-dependent enzymes in non-conventional media and their effects on the reaction mechanism have recently been published. As already mentioned above, especially the behaviours of benzaldehyde lyase from *Pseudomonas fluorescens* (*Pf*BAL^5^,^24^) and benzoylformate decarboxylase from *Pseudomonas putida* (*Pp*BFD) in monophasic buffer-organic solvent mixtures have been examined.24a However, these studies only focused on the syntheses of benzoin and HPP, respectively.

In the present study, we report on the first comprehensive analysis of six representatives of the well-characterized group of ThDP-dependent enzymes in combination with 13 organic solvents of different concentrations. We provide one of the most extensive and reliable databases published so far, which allows us to identify general trends of different organic cosolvents not only with respect to the stereoselectivity but also the product range (chemoselectivity) of these enzymes. The results range from almost no effect on both stereo- and chemoselectivity to significant alterations of one or even both selectivities. As a consequence, we were able to elucidate size- and polarity-dependent effects of the different solvents which can be explained by solvent binding to the active site.

## Results

**Figure 3:**
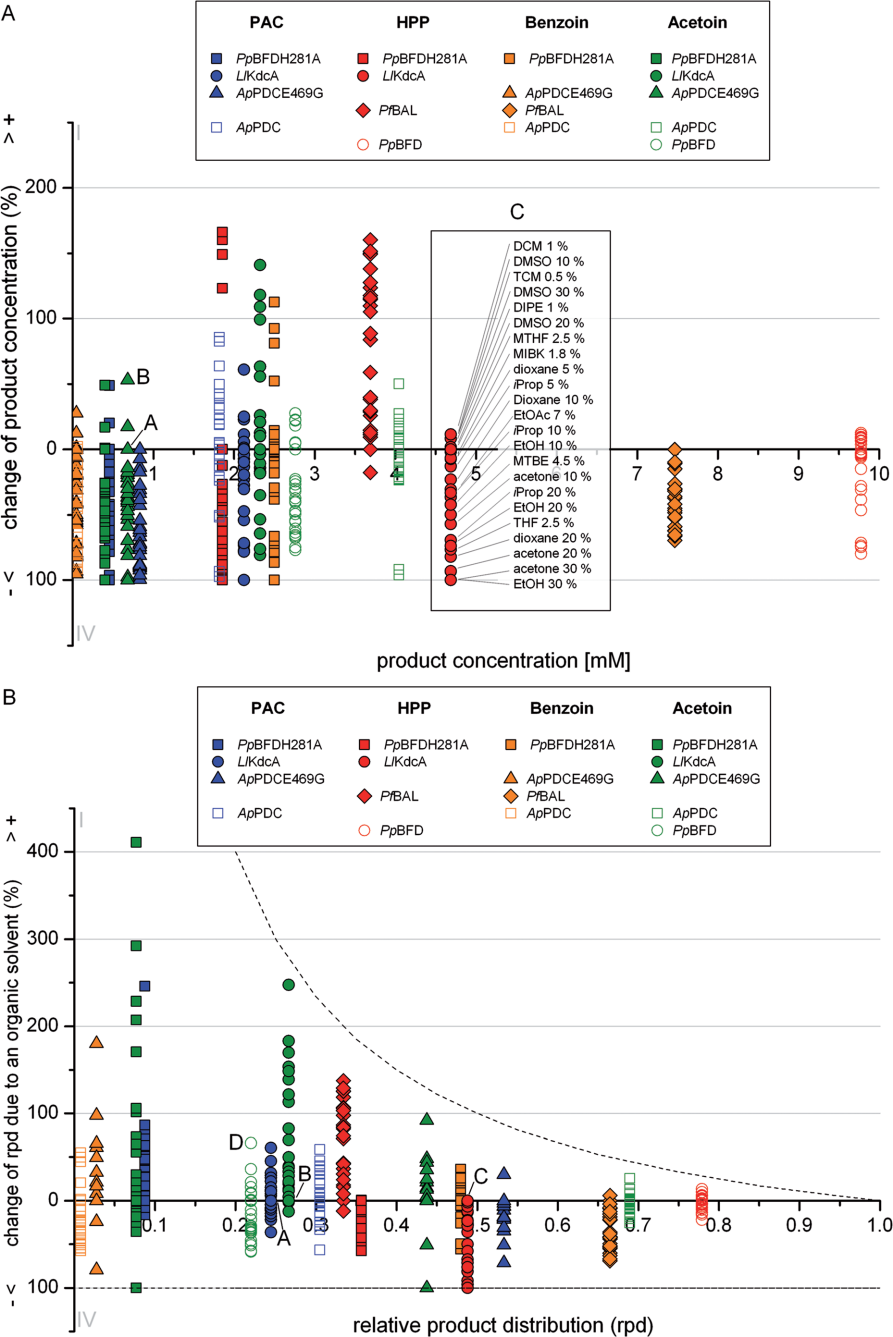
(A) Correlation between the change of the final product concentrations (mM) after 24 h influenced by the addition of organic solvents relative to the final product concentrations in aqueous buffer without solvent addition is shown. (B) Correlation between the change of relative product distribution (rpd) and rpd in aqueous buffer without solvent addition is presented. The symbols represent the respective enzymes and the colors refer to the respective products. Every data point represents the influence of one organic solvent in one defined concentration (for details of every single organic solvent and its effect on each enzyme see the more extensive graphs in the Supporting Information). Data points in the first quadrant refer to a higher final product concentration (A) or relative product distribution (B) after 24 h relative to the control in buffer, whereas data points in the fourth quadrant relate to reduced product concentration (A) or reduced relative product distribution (B) due to the cosolvents. A–B (A) and A–D (B) refer to examples discussed in the text. Box C (A) shows once as example the solvents which induce the respective change in HPP production of *Ll*KdcA. The dashed lines in the 1^st^ quadrant and at 100% in the 4^th^ quadrant give the limitations in which values due to the percentage plotting may appear. *Substrate concentrations:* 18 mM benzaldehyde (all enzymes) and 18 mM (*Ap*PDC, *Ap*PDCE469G, *Pf*BAL) or 180 mM (*Ll*KdcA, *Pp*BFD, *Pp*BFDH281A) acetaldehyde.

**Figure 4:**
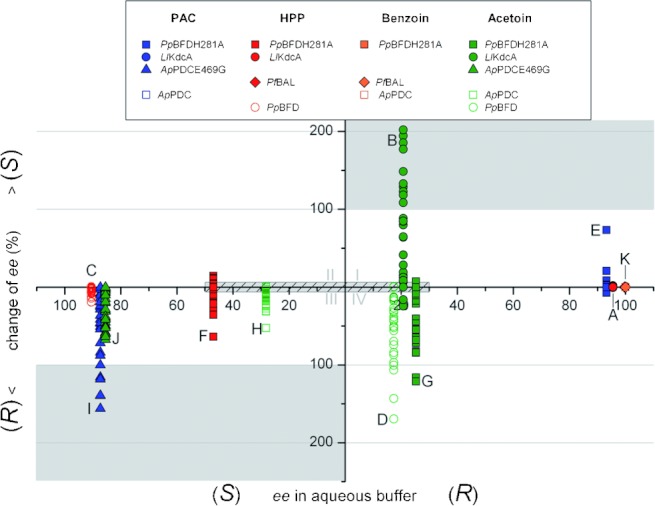
Correlation between the influence of organic solvents after 24 h on the enantiomeric excess (*ee*) relative to the *ee* in aqueous buffer (given on the x-axis). The symbols represent the respective enzymes and the colors refer to the respective products. Each data point represents the change induced by one solvent in one concentration (for details, see Supporting Information, Figure S2 to Figure S7). The grey areas mark results where stereoinversion was observed. On the x-axis, the *ee* of the buffer control in aqueous media is plotted in a range between 100% (*S*) and 100% (*R*). In the vertical direction, the change in the *ee* relative to the buffer control is plotted. The letters A–K refer to examples discussed in the text. The hatched grey area is addressed in the discussion.

To analyze the influence of organic cosolvents on the behaviour of ThDP-dependent enzymes in detail, we performed about 400 independent batch reactions, which were analyzed by more than 1100 HPLC and GC runs. It would not be appropriate to discuss all these results in detail. A collection of the complete results and thus the influence of every single organic solvent on enzyme behavior can be found in the supporting information. In order to deduce common effects from this extensive data set, we combine the essential values graphically (Figure 3 and Figure 4).

### Chemoselectivity

To study possible changes in the productivity and chemoselectivity of ThDP-dependent enzymes, 13 different organic solvents in different concentrations were added to six enzymes and the concentration of all the products formed was determined after 24 h by HPLC and GC analyses (for detailed results see the Supporting Information). The complex results are summarized in Figure 3, A.

In this plot, each of the four possible products is considered independently. An example is given in order to help the reader understand this highly complex diagram. Acetoin is produced by *Ap*PDCE469G catalysis in a concentration of 0.68 mM (Figure 3, A, point A on the x-axis). Upon addition of an organic solvent (here 20 vol% DMSO), productivity was increased to 1.05 mM. The relative increase of 54.4% of the product concentration is plotted against the starting concentration of 0.68 mM (Figure 3, A, point B in the 1^st^ quadrant). The second example to understand this graph is the production of HPP by *Ll*KdcA. For that combination, all solvents are correlated to their influence (Figure 3, A, C) to demonstrate, that every data point represents the influence of one organic solvent in one concentration. Reduced product concentrations, which can be explained by reduced activities and/or stabilities in the presence of organic solvents, are also often detectable, leading to a negative change of the final product concentration.

Taking all the results into account, in 26.1% of cases we found increased productivity upon addition of an organic solvent, whereas in 73.9% of cases solvent addition led to a reduction of enzyme productivity. In the buffer control without organic cosolvent, the conversion of benzaldehyde in the given set-up was in the best case 89% with *Pf*BAL and in the worst case 5% for *Ap*PDCE469G after 24 h.

For a better understanding of how the solvent influences chemoselectivity, changes in the relative product distribution (rpd) are given in Figure 3, B (a stepwise guideline to read this plot can be found in the Supporting Information). The rpd is defined as the concentration of one product in relation to the sum of the concentrations of all observed products in the respective carboligation reaction {[product A]/([product A]+[product B]+[product C]+[product D])}.

Although Figure 3, A and Figure 3, B are complex, they very clearly show the great influence of organic solvents on enzyme productivity and chemoselectivity. The most pronounced effects for each enzyme are discussed in the following.

*Ll*KdcA accepts aliphatic and aromatic donor and acceptor aldehydes comparatively well and is thus very unselective with respect to its chemoselectivity. Consequently, it is one of the enzymes in our study with the broadest product range, including HPP, PAC and acetoin.^12^ Only the bulky benzoin, derived from the ligation of two molecules of benzaldehyde, is not accessible. In the absence of cosolvents, PAC and acetoin are formed in almost equal amounts under the chosen conditions [18 mM benzaldehyde, 180 mM acetaldehyde, rpd=0.24 (PAC, Figure 3, B, A), rpd=0.27 (acetoin, Figure 3, B, B)], while HPP production was higher by a factor of 2 (rpd=0.49, Figure 3, B, C). As demonstrated in Figure 3, A, *Ll*KdcA represents one of the examples where some organic cosolvents cause an increased product concentration. This is true for acetoin and PAC, whereas the productivity for HPP was reduced by almost all organic solvents (Figure 3, B). Beneficial cosolvents for acetoin synthesis with *Ll*KdcA are: 10 vol% ethanol (EtOH), 10–30 vol% acetone, 10–30 vol% DMSO, 10 and 20 vol% isopropyl alcohol (*i*Prop), 5 and 10 vol% dioxane, 1 vol% dichloromethane (DCM), 1 vol% diisopropyl ether (DIPE), 7 vol% ethyl acetate (EtOAc), 0.5 vol% trichloromethane (TCM) and 1.8 vol% methyl isobutyl ketone (MIBK). If one solvent was tested in different concentrations, the improving effect often increased with rising concentration. In the presence of these cosolvents, the final product concentration was enhanced from 2.3 mM in the buffer control to 2.9–25.4 mM. Moreover, the production of PAC was enhanced from 2.1 mM in the buffer control to 2.2–4.7 mM by 0.5 vol% TCM, 1 vol% DCM, 10 vol% EtOH, 20 vol% *i*Prop, 10 and 30 vol% DMSO, 5 and 10 vol% dioxane and 10 vol% acetone (Figure 3 A, Supporting Information, Figure S2).

In conclusion, in the presence of the organic solvent a clear shift was observed toward products derived from the smaller aldehyde (acetaldehyde) donor, whereas HPP, which is derived from benzaldehyde as the donor, is reduced.

With benzoylformate decarboxylase from *Pseudomonas putida* (*Pp*BFD) only the production of HPP (9.8 mM, rdp 0.78) and acetoin (2.8 mM) was observed under the chosen conditions (18 mM benzaldehyde, 180 mM acetaldehyde), whereas benzoin, although found under different reaction conditions in previous work,^25^ and PAC could not be detected. With a few exceptions, the productivity for both products was reduced by the cosolvents (Figure 3, A, Supporting Information, Figure S3). Regarding the relative product distribution (rpd), only minor effects are visible for the production of HPP, while the rpd of acetoin varies in the range of −58% to +65% relative to the buffer control (rpd=0.2, Figure 3, B, Supporting Information, Figure S3). The highest rpd for acetoin was observed in the presence of 30 vol% *i*Prop (Figure 3 B, D, +65% to a value of rpd=0.36). With respect to the distribution of the data for HPP and acetoin, the product with the higher rpd value in buffer (HPP, 0.78) is less prone to reduction by organic solvents, since the influence of organic solvents and thus the reduction of the product with the lower rpd (acetoin, 0.22) is greater (Figure 3, B).

The variant *Pp*BFDH281A has an enlarged active site relative to the wild-type enzyme *Pp*BFD and was designed for improved benzoin synthesis.^10^ In the chosen experimental setup (18 mM benzaldehyde, 180 mM acetaldehyde), the enzyme catalyzes the synthesis of all four possible products of the mixed carboligation, thus being very unselective. In aqueous buffer, all respective products were obtained in a concentration <3 mM (which is related to <16% conversion of benzaldehyde to each product). As has already been found for the wild-type enzyme, productivity is in most cases reduced by the organic cosolvents (Figure 3, A). However, with 10 vol% DMSO, 0.5 vol% TCM and 1 vol% DCM, the product concentration of HPP can be slightly enhanced. Additionally, 10–20 vol% DMSO, 10 vol% EtOH, 10 vol% *i*Prop, 10 vol% acetone, 1 vol% DCM, 0.5 vol% TCM and 1 vol% DIPE enhance the product concentration of benzoin to 3.7 mM after 24 h of reaction (Figure 3, A, Supporting Information Figure S4). Again we observed an enhancement of the rpd for acetoin and PAC, which contain acetaldehyde as the donor, while the productivity for benzoin and especially HPP (both originating from benzaldehyde as the donor) is reduced (Figure 3, B). Consequently, the addition of organic additives seems to hamper the binding of the more bulky substrate benzaldehyde to the donor binding site more than the acceptor binding site, yielding smaller 2-hydroxy ketones.

Acetoin, PAC and traces of benzoin were produced by catalysis of *Ap*PDC under the selected reaction conditions (18 mM benzaldehyde, 18 mM acetaldehyde) without solvent addition. In the buffer control, acetoin accumulates to a concentration of 4 mM after 24 h, which can be increased to 5 mM in the presence of 30 vol% acetone. Although some solvents reduce the productivity, an enhancement of the PAC production from 1.8 mM in the buffer control to 3.1 mM is also possible, for example with 5 vol% MTHF. The other solvents which enhance the PAC production are DMSO, dioxane, EtOAc and *i*Prop (Supporting Information, Figure S5). Compared to the other products, the concentration of benzoin is very low (0.06 mM) and is further decreased by the addition of most solvents (Figure 3, A, Supporting Information, Figure S5). Regarding the rpd values for the buffer controls in comparison to each other, taking into account the reduction of the rpd values in the vertical direction, it seems that the production of acetoin (rdp=0.69) is more resistant to the addition of organic solvents than the production of PAC (rpd=0.3) and benzoin (rpd<0.01) (Figure 3, B).

In previous work, an *Ap*PDC variant with a mutation of position 469 from glutamate (E) to glycine (G) was successfully prepared in order to generate an *S*-selective enzyme for the production of (*S*)-PAC and derivatives thereof.11d The productivity of *Ap*PDCE469G is reduced by almost all cosolvents relative to the control in aqueous buffer under the chosen conditions (18 mM benzaldehyde, 18 mM acetaldehyde, Figure 3, A), except for 10 vol% and 20 vol% DMSO, which increase the final product concentration for acetoin with this variant (Figure 3, A, Supporting Information, Figure S6). Although the overall productivity is often reduced, we observed a shift to the smaller product by some of the cosolvents, because the rpd for acetoin is enhanced at the expense of PAC (Figure 3, B).

*Pf*BAL, a highly active carboligase preferring benzaldehyde as the donor aldehyde,13b catalyzes the formation of HPP and benzoin. Some studies have already been conducted for this enzyme analyzing the influence of organic solvents. It was found that the stability of *Pf*BAL can be enhanced in the presence of DMSO.^5^ In the buffer control of the present experimental set-up (18 mM benzaldehyde, 18 mM acetaldehyde), 3.7 mM HPP and 7.5 mM benzoin were observed after 24 h. As demonstrated in Figure 3, A, EtOH, *i*Prop, acetone, dioxane and DMSO enhance the product concentration of HPP after 24 h to values of up to 8.9 mM, 8 mM, 9.3 mM, 9 mM and 8.1 mM, respectively (see also Supporting Information, Figure S7). For DMSO the enhancement is proportional to the increase of its concentration. Moreover, all cosolvents (except TCM) enhance the rpd for HPP and reduce it for benzoin (Figure 3, B, Supporting Information, Figure S7).

In conclusion, we observed a general trend concerning the influence of organic cosolvents on the chemoselectivity of the six ThDP-dependent enzymes. Regarding the final product concentration of a certain product relative to all products formed (described as rpd in Figure 3, B), it becomes obvious that the lower the chemoselectivity of the enzyme (and therefore the less selective the enzyme is for the formation of only one product, represented by a low rpd value), the higher is the decrease of rpd by the organic solvent. The second obvious trend of the organic cosolvents is a shift of the chemoselectivity towards the smaller 2-hydroxy ketone in most cases, except for the PAC production catalyzed by *Ll*KdcA and *Pp*BFDH281A, where HPP is reduced while both PAC and acetoin rpd values are increased. Here a trend to a smaller donor aldehyde seems to occur. The other exceptions are *Pp*BFD, which shows a variation of rpd of acetoin and HPP, but no shift to acetoin and *Ap*PDC, where no shift in any direction was detectable. Although the productivity is often reduced by the cosolvents, there are also some examples of increased productivity, for example, acetoin formation by *Ll*KdcA, HPP formation by *Pf*BAL and PAC formation by *Ap*PDC. These increased product concentrations can be induced by different solvents, but DMSO is always one of them. The only exception to this rule is *Pp*BFD, where none of the solvents were able to increase any product concentration.

### Stereoselectivity

The influence of organic cosolvents on the stereoselectivity of the mixed carboligation reaction catalyzed by ThDP-dependent enzymes was of special interest to determine the potential of medium engineering on asymmetric syntheses. The results of the stereoselectivity studies are summarized in Figure 4.

It can be clearly seen that the variations in stereoselectivity due to the influences of the cosolvents are very different, depending on the enzyme and its stereoselectivity observed in the buffer control. Changes for the respective product were observed in the direction of the *R*-enantiomer (quadrants III and IV) as well as the *S*-enantiomer (quadrants I and II). All data in quadrants I and III above 100% show stereoinversion (gray areas), due to the presentation of percentage changes. Data in quadrants II and IV show values with improved stereoselectivity for the respective products. Here, values above 100% relate to a more than doubled enantiomeric excess (*ee*) for the respective enantiomer relative to the control in buffer (x-axis). The most pronounced effects on the stereoselectivity for each enzyme are discussed in the following:

*Ll*KdcA catalysis yields the mixed products PAC and HPP with very good *ee*s [PAC >99% *ee* (*R*), HPP 95% *ee* (*R*)]. This selectivity is not changed in the presence of organic solvents (Figure 4, A; Supporting Information, Figure S2). In contrast, the stereoselectivity for acetoin in buffer is low [19% *ee* (*R*)]. Although it was not possible to increase the selectivity of *Ll*KdcA for acetoin in the *R*-direction, the addition of, for example 10–30 vol% acetone resulted in a proportional increase of the *S*-enantiomer concentration yielding a maximal *ee* of 21% (*S*) with the highest concentration (Figure 4, B; Supporting Information, Figure S2).

A similar trend was observed for *Pp*BFD. The enzyme stereoselectivity for the rather selectively produced product (*S*)-HPP (*ee*=91% in buffer) is only slightly affected compared to the unselectively produced acetoin, where the *ee* of (*R*)-acetoin (17% *ee* in buffer) was more than doubled by some of the cosolvents, for example, 30 vol% *i*Prop [46% *ee* (*R*)] (Figure 4, C and D; Supporting Information, Figure S3).

The variant *Pp*BFDH281A is the only enzyme in this study which catalyzes the formation of all four possible products (Figure 1) in sufficient amounts to allow the determination of stereoselectivity for all of them. (*R*)-Benzoin is produced in an enantiomerically pure form (*ee* >99%) and the *ee* is not affected by any cosolvent tested. (*R*)-PAC is also produced with a good *ee* of 93%. Its stereoselectivity is shifted toward the *S*-product by, for example, 20 vol% dioxane [25% *ee* (*R*)] (Figure 4, E). A similar effect was observed for (*S*)-HPP, where the initial *ee* of 47% in buffer was decreased to 17% *ee* (*S*) by, for example, 7 vol% EtOAc (Figure 4, F). For the smallest product, (*R*)-acetoin, the stereoselectivity was significantly enhanced from 25% to 56% *ee* (*R*) by 7 vol% EtOAc (Figure 4, G; Supporting Information, Figure S4).

In comparison to the other enzymes, the effect of organic solvents on *Ap*PDC stereoselectivity is limited. PAC [*ee* >99% (*R*) in buffer] and benzoin [*ee* >99% (*R*) in buffer] are produced with high *ee*s and again no influence of cosolvent addition can be detected. The situation is different for (*S*)-acetoin formation where the initial *ee* of 28% in buffer was reduced to 13% (*S*) in the presence of 5 vol% tetrahydrofuran (THF) (Figure 4, H; Supporting Information, Figure S5).

Compared to the wild-type enzyme, the stereoselectivity of the *S*-selective variant *Ap*PDCE469G is very sensitive to the addition of organic solvent. The variant catalyzes the synthesis of (*S*)-PAC (87% *ee*) in buffer. The addition of, for example, 0.5 vol% TCM led to stereoinversion yielding predominantly the *R*-enantiomer [49% *ee* (*R*)] (Figure 4, I; Supporting Information, Figure S6). This example shows one of the most pronounced effects of this study on stereoselectivity shifts by the addition of an organic solvent. The same trend was observed with acetoin, which was formed with an *ee* of 85% (*S*) in buffer, but with only 28% *ee* (*S*) in the presence of 30 vol% EtOH (Figure 4, J; Supporting Information, Figure S6). For the selectivity of the *Ap*PDCE469G often an impact was found, which is commensurate to an increase of cosolvent concentration.

In contrast, the stereoselectivity of the highly selective *Pf*BAL is not affected by the different organic solvents at all. (*R*)-Benzoin and (*R*)-HPP are formed in an enantiomerically pure form (>99% *ee*), independent of any added cosolvent (Figure 4 K; Supporting Information, Figure S7).

In summary, it can be stated that in all cases where the stereoselectivity of the products was already very high (approximately *ee* >95%) in buffer, the organic solvents have no effect on stereoselectivity. In contrast, for products which are formed less selectively, inversions from *S*- to *R*-selectivity were observed, whereas it seems to be impossible to improve an initial *S*-selectivity. However, starting from an *R*-selectivity both, the inversion to the *S*-enantiomer in excess or the improvement of that *R*-selectivity were possible.

Taking the results of the overall product concentrations (Figure 3, A) and stereoselectivity shifts (Figure 4) into account, it becomes obvious that in some cases a change in stereoselectivity is accompanied by reduced overall activity. In such cases, it is most likely that one of the pathways [(*R*)- or (*S*)-, Figure 2] is blocked, but that the other pathway is still accessible. In that case, stereoselectivity is shifted due to selective activity reduction.

## Discussion

Some general trends can be concluded from the behaviour of the different ThDP-dependent enzymes in the presence of water-miscible organic solvents. (i) The chemoselectivity of the enzymes seems to be more resistant to the addition of organic solvents if the enzyme is highly selective for the respective product in aqueous buffer (Figure 3, B). (ii) Solvent-induced shifts in chemoselectivity are often related to a shift to the respective smaller 2-hydroxy ketone (Figure 3, B). (iii) In all the reactions where an increased product concentration was detectable, DMSO was always one of the improving solvents. (iv) The stereoselectivity of the enzymes is not influenced by organic cosolvents if the stereoselectivity in buffer for the respective product is high (*ee* >95%, Figure 4). This characteristic is in accordance with the observations for solvent-induced chemoselectivity shifts [see trend (i)]. (v) In the case of acetoin, inversion from *S*- to *R*-selectivity was observed (Figure 4). (vi) It is impossible to further improve an initial *S*-selectivity by organic cosolvents (Figure 4).

Possible reasons for the strong influence of organic solvents on ThDP-dependent enzymes are as follows. Firstly, an influence of the solvents on the three-dimensional structure and on the flexibility of the active site is possible. For example, only small changes in the three-dimensional structure of the active site would change the spatial constraints enabling the substrates to be arranged in a defined position in the active site thus affecting both chemo- and stereoselectivity. These structural shifts would additionally influence the kinetic parameters of the enzymes. Furthermore, the organic solvent itself might influence the affinity of the substrate to the active site as well as the kinetic parameters of the different steps in the catalytic cycle (Supporting Information, Figure S1), transition state formation and/or product formation and release. On the one hand, this can be explained by changes in the hydrophobicity/hydrophilicity of the surrounding media. Small organic molecules might be able to enter the active site and thus block parts of it, which would result in changes in the overall activity and selectivity. It has already been shown that a cosolvent was able to enter the active site of *Pf*BAL and attach to hydrophobic patches.^26^ On the other hand, the solubility of the substrate, intermediates and products can be enhanced or impaired by shifting kinetic parameters, equilibria and final product concentrations.

There are enthalpic differences between the enantiomers during catalysis.^27^ The difference in activation free energy between the enantiomers [ΔG=−RTln(100+*ee*)×(100−*ee*)^−1^] can be influenced by structural or kinetic shifts, causing *ee* changes.

In the following paragraphs, the different possibilities will be analyzed and discussed.

### Effects on Enzyme Flexibility

**Figure 5:**
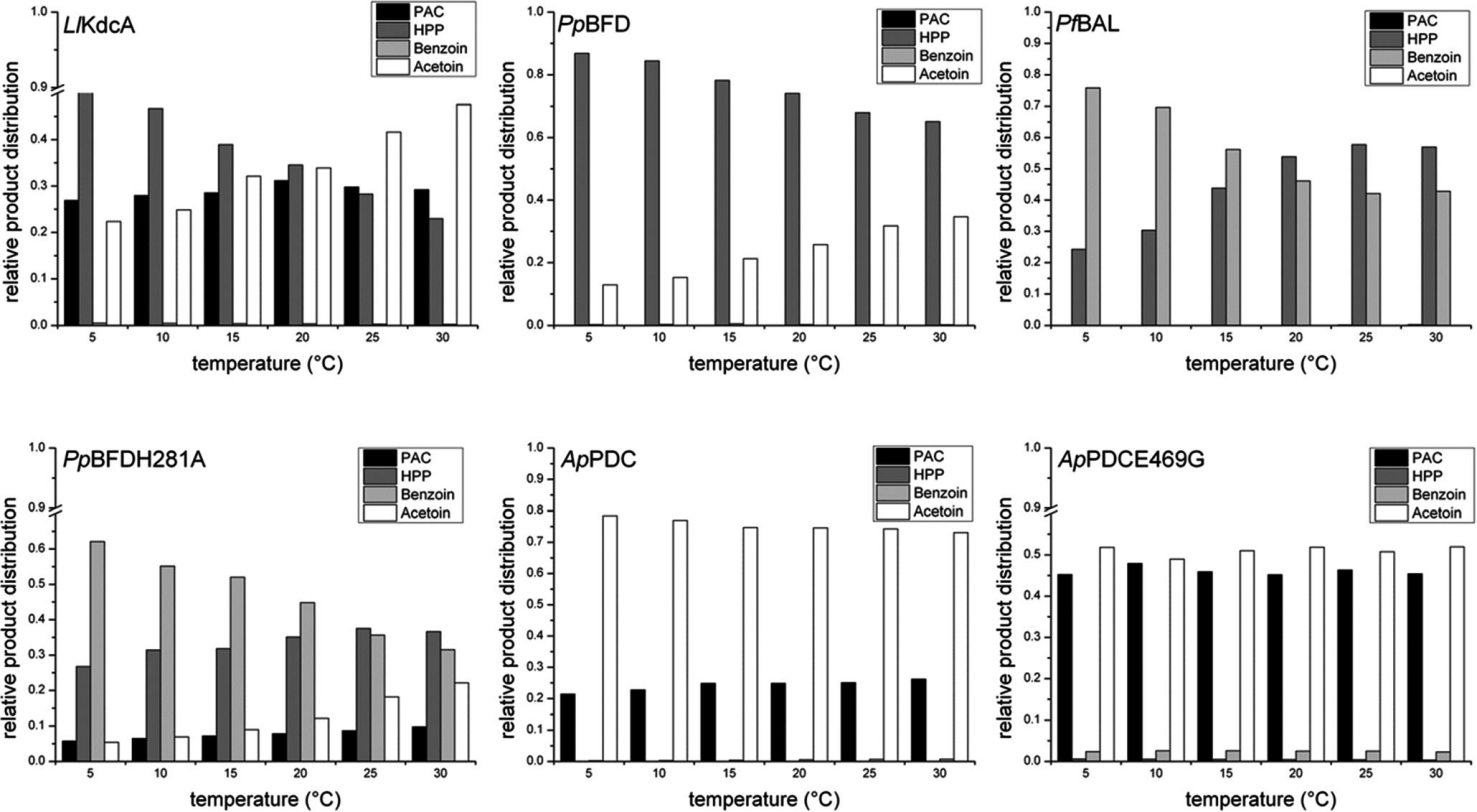
Influence of different reaction temperatures on the chemoselectivity of the six investigated ThDP-dependent enzymes catalyzing the carboligation of benzaldehyde and acetaldehyde. *Substrate concentrations:* 18 mM benzaldehyde (all enzymes) and 18 mM (*Ap*PDC, *Ap*PDCE469G, *Pf*BAL) or 180 mM (*Ll*KdcA, *Pp*BFD, *Pp*BFDH281A) acetaldehyde.

Concerning the influence of cosolvents on enzyme flexibility, several diverging results can be found in the literature. Examples were given showing that increased flexibility decreases stereoselectivity. There are also examples where no shifts in selectivity occurred due to rising flexibilities, at least in pure organic solvents.^23^ This seems to vary depending on the biocatalyst, its structure and its reaction mechanism. At the same time, enhanced flexibility makes the enzyme more prone to unfolding^20^ and reduces the overall activity. To shed light on this topic with respect to our ThDP-dependent enzymes, the carboligation of benzaldehyde and acetaldehyde was studied at different temperatures in the absence of cosolvents. This approach was used as an alternative to increase enzyme flexibility. The results are presented in Figure 5.

The results demonstrate that a temperature rise has a significant impact on enzyme chemoselectivity. In the case of *Ll*KdcA, the chemoselectivity is shifted from HPP to acetoin with increasing temperature. The same holds for *Pp*BFD. In the case of the variant *Pp*BFDH281A the synthesis of benzoin decreases with increasing temperature, whereas acetoin and HPP increase. Also in the case of *Pf*BAL, we observed a shift from benzoin to HPP. Altogether, an increased reaction temperature resulted in a shift from the larger 2-hydroxy ketone to the smaller one. Exactly the same trend was observed with organic cosolvents in the case of *Pf*BAL, *Pp*BFD and *Ll*KdcA. By contrast, the chemoselectivity of *Ap*PDC and the variant *Ap*PDCE469G is not temperature-sensitive. This might be due to the fact that these are the only enzymes in the series tested which strongly prefer acetaldehyde to benzaldehyde as the donor. The effect of higher molecular movements due to the elevated temperature is therefore less pronounced, since these two enzymes are not able to accept benzaldehyde instead of acetaldehyde as the donor. However, the other enzymes tested have two alternative donor substrates since they accept acetaldehyde as well as benzaldehyde. And for them a clear tendency can be seen from the larger to the smaller donor aldehyde with increasing temperature.

There are several possible explanations for the similar effects observed with increased temperature and organic cosolvents. On the one hand, the organic solvents might affect the flexibility of the enzymes as the temperature does. It might therefore be possible that the increased flexibility only allows the production of the smaller products because of steric hindrances due to the increased molecular movements of side chains in the active site. On the other hand, the organic solvents as well as the temperature might have an impact on the microreaction rates of the reaction and the affinity of the substrates, which might result in the preferred synthesis of one product to the other. Furthermore, the solubility of the different compounds of the reaction is influenced by the organic solvents and the temperature.

### Effects on the Solubility of Substrates, Intermediates and Products

Organic cosolvents influence the solubility of all compounds in the reaction mixture, especially if aromatic compounds such as benzoin are involved. This might influence the kinetic parameters. The temperature-sensitive chemoselectivity of *Pf*BAL is a good example to study this effect. Previous investigations strongly suggested that the BAL-catalyzed formation of HPP involves the intermediate formation of benzoin from benzaldehyde. Benzoin is subsequently cleaved again, yielding ThDP-bound benzaldehyde which then reacts with acetaldehyde as an acceptor yielding HPP.2b As benzoin shows a very low solubility in aqueous media (<1 mM), it precipitates during the course of the reaction. As a consequence, the concentration of benzoin as one donor-delivering compound is reduced. Thus, besides flexibility changes, the result of a temperature increase on *Pf*BAL might be an indicator for the influence of temperature on the available substrate concentration and thus on the reaction kinetics. Both elevated temperatures as well as organic cosolvents increase the solubility of benzoin and thus the production of HPP relative to benzoin. However, this cannot be the whole explanation, since only *Pf*BAL with its special reaction cycle2b is prone to solubility limitations. No solubility limitations were observed with the other tested enzymes, but equilibrium shifts are possible.

### Direct Interaction of Solvents with the Active Site

Furthermore, a direct interaction of the solvent molecules with the enzyme active sites can be assumed. The active site of ThDP-dependent enzymes is located in a cleft between two monomers of the homodimer. Apart from *Ll*KdcA, in all other cases two homodimers associate to form a homotetramer. ThDP, as the catalytically active species, is located at the bottom of the active site, which is connected to the enzyme surface by a channel. As the active sites of all enzymes contain several hydrophobic residues, a direct interaction with the solvent molecules can be assumed. In Table 1, we compare the solvent-excluded volumes of the substrates and products of the carboligation reaction to the different solvents.

**Table 1:** Overview of the substrates and products of bimolecular carboligation (highlighted in italics) and the implemented organic solvents, sorted according to their calculated solvent-excluding volume (calculated by ChemDraw for Excel Add-In). The concentration range is also given in which the organic solvents were used (exact concentration values are given in the Supporting Information; values in parentheses are those used for Figure 6)

Name	% (v/v)	log P	Solvent-excluded volume [Å^3^]
*acetaldehyde*			*42.7*
EtOH	5–30 (30)	−0.28	48.3
DCM	1 (1)	1.25	49.4
acetone	2–30 (10)	−0.24	59.1
TCM	0.5 (0.5)	1.94	59.1
DMSO	5–30 (20)	−1.35	61.5
*i*Prop	5–30 (20)	0.05	62.5
THF	2.5–5 (2.5)	0.34	71.2
dioxane	5–30 (10)	−0.35	79.0
*acetoin*			*80.5*
EtOAc	7 (7)	0.85	82.7
MTHF	2.5–5 (2.5)	1.39	85.0
MTBE	4.5 (4.5)	0.94	86.2
*benzaldehyde*			*94.6*
MIBK	1.8 (1.8)	1.15	105.5
DIPE	1 (1)	1.52	106.6
*PAC*			*127.3*
*HPP*			*128.9*
*benzoin*			*175.7*

It can be clearly seen that as a function of their size all the solvents should be generally able to access the active sites.

As described in the introduction, the stereoselectivity of ThDP-dependent enzymes can be mainly explained by steric factors influencing the relative position of donor and acceptor in the active site prior to C–C bond formation (Figure 2).

Especially for *Ap*PDCE469G, extensive structure-function investigations have been conducted explaining chemo- and stereoselectivity in bimolecular carboligation reactions.11d In this enzyme, a very large *S*-pocket was designed where the side chain of benzaldehyde can be optimally stabilized in an antiparallel manner and at a reactive distance to the ThDP-bound donor aldehyde (acetaldehyde). None of the other enzymes possesses such a large pocket for *S*-selective carboligation.

The fact that it is almost impossible to improve initial *S*-selectivity by the addition of cosolvents (Figure 4) may be explained by a blockade of the *S*-pocket by small organic solvents. If this is the case, the residue of the acceptor aldehyde would no longer be able to enter the *S*-pocket and the *S*-pathway would be blocked, resulting in an increase of the *R*-enantiomer. All of the tested organic solvents except for MIBK and DIPE are smaller than the substrate benzaldehyde (Table 1) and therefore theoretically able to enter and block the *S*-pocket in an enzyme capable of catalyzing the formation of (*S*)-PAC.

**Figure 6:**
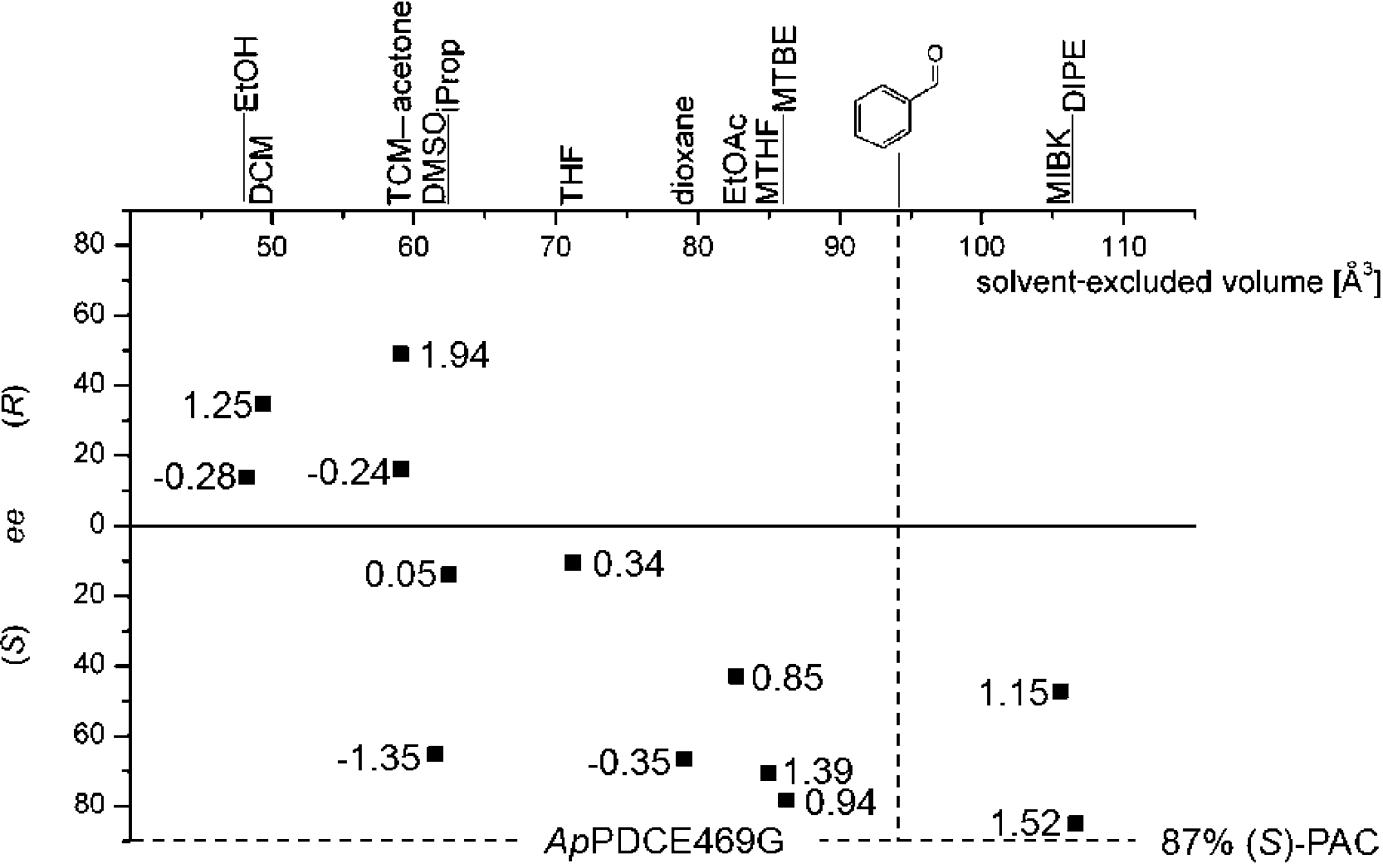
Influence of different organic cosolvents (concentrations are given in parenthesis in Table 1, sorted according to their size-excluded volume [Å^3^]) on the stereoselectivity of PAC formation catalyzed by *Ap*PDCE469G. Respective log P values are indicated next to the data points. In buffer, (*S*)-PAC is obtained with an *ee* of 87% (horizontal dashed line, compare Figure 4). Also the size of benzaldehyde is given (vertical dashed line), because the phenyl side chain has to fit into the *S*-pocket to yield (*S*)-PAC.

Since *Ap*PDCE469G has the largest *S*-pocket and is the only known biocatalyst for the production of (*S*)-PAC,11d any blockade effects by organic solvents at this part of the active site should be detectable by a decrease of the *S*-selectivity. It was precisely this shift from highly *S*-selective product formation towards *R*-product formed in excess that was measured. In order to further analyze the effects, we plotted the changes observed in the stereoselectivity of *Ap*PDCE469G from (*S*)-PAC to (*R*)-PAC against the size of the solvents inducing these changes (Figure 6).

Figure 6 shows a clear correlation between the size of the solvent and its influence on the stereoselectivity of the *Ap*PDCE469G-catalyzed (*S*)-PAC formation: smaller solvents reduce the initially good *ee* of 87% (*S*) (in buffer) more strongly than larger ones. A stereoinversion is observed in the presence of EtOH, DCM, acetone and TCM. The smaller the solvent, the higher is the possibility of an occupied *S*-pocket. This result in a blockade of the antiparallel arrangement of both substrates in the active site and thus the parallel arrangement yielding *R*-product is preferred (Figure 2). A similar size-dependent influence on the stereoselectivity was described earlier for lipases in neat organic solvents.^27^ However, the size of the solvent is not the only correlation. There are some pairs of organic solvents of almost the same size, which have a different impact on enzyme selectivity. Here, the second important fact is the polarity, namely log P, of the solvents, where P is the partition coefficient of the solvent between octanol and water. No correlation became obvious by plotting only the log P against the *ee* shifts. However, if both factors are included, a correlation of the size of the solvent and its log P becomes clearly visible. An example is the case of EtOH and DCM, which have almost the same solvent-excluded volume. However, DCM which is less polar than EtOH has a stronger impact on the stereoselectivity. The same is true for the following pairs: acetone and TCM, DMSO and *i*Prop, THF and dioxane, and EtOAc and dioxane. It can be concluded that the less polar solvent can better accumulate in the non-polar *S*-pocket, since it is mainly built of hydrophobic amino acid side chains^4^.

In the case of (*S*)-PAC formation, it can be assumed that there is always a competition between the organic solvent and the benzaldehyde for the binding positions in the *S*-pocket. Taking into account the molar proportion of the different compounds, it becomes obvious that in a synthesis reaction employing 18 mM benzaldehyde and, for example, 8.24 mol/L EtOH (=30 vol%, Supporting Information, Table S1) the solvent is in a 500-fold excess. Even the lowest concentration of 0.03 mol/L TCM (=0.5 vol%, supporting information, Table S1), is still present in a 1.7-fold excess relative to the competing benzaldehyde.

The second piece of evidence for the blocking theory is provided by another *S*-selective reaction catalyzed by the enzyme *Pp*BFD, where acetaldehyde acts as the acceptor of the reaction yielding acetoin and HPP as products.11b Again, the initial *S*-selectivity for HPP in buffer (*ee* 90.7%) cannot be improved by any of the cosolvents. However, in contrast to PAC production by *Ap*PDCE469G, we observed no significant shifts towards the *R*-enantiomer in the presence of the cosolvents (Supporting Information, Figure S3). Since the *S*-pocket is much smaller in *Pp*BFD, only very small solvent molecules would theoretically be able to enter. However, in our set-up there is no solvent smaller than acetaldehyde (Table 1), which explains the constant stereoselectivity of *Pp*BFD for the synthesis of (*S*)-HPP (Supporting Information, Figure S9).

**Figure 7:**
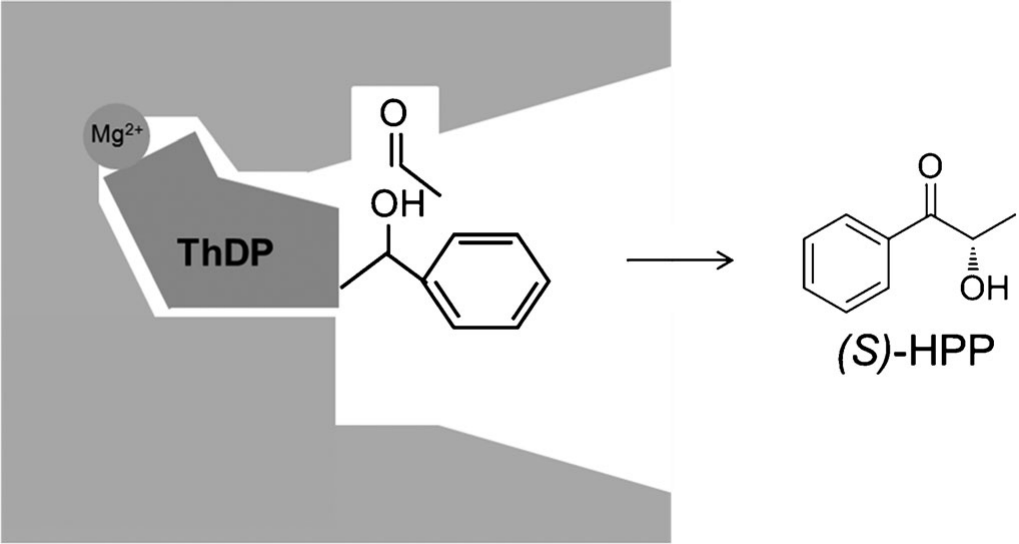
Schematic representation of the active site of *Ll*KdcA showing the situation prior to C–C bond formation. The *Si*-site attack of acetaldehyde as an acceptor may offer an alternative route to (*S*)-HPP (compare Figure 2).

**Figure 8:**
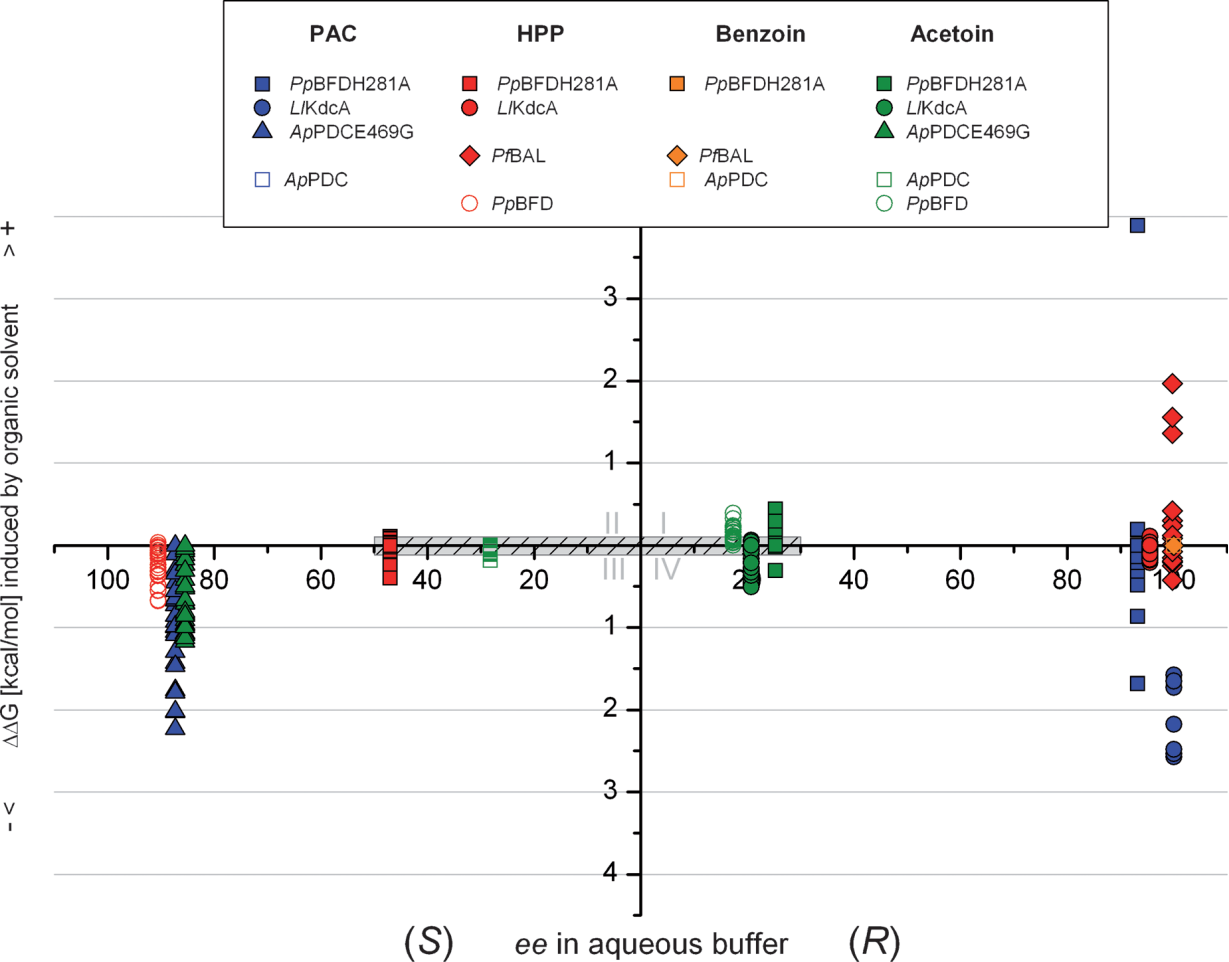
Correlation between the influence of organic solvents after 24 h on the enantiomeric excess (*ee*), represented by ΔΔG, relative to the *ee* in aqueous buffer (given on the x-axis). The symbols represent the respective enzymes and the colors refer to the respective products. Each data point represents the change induced by one solvent in one concentration (for details see the Supporting Information, Figure S2 to Figure S7). On the x-axis, the *ee* of the buffer control in aqueous media is plotted in a range between 100% (*S*) and 100% (*R*). In the vertical direction, the change of the *ee*, represented by ΔΔG, relative to the buffer control is plotted. The values are calculated with ΔG=−RTln(100+*ee*)×(100−*ee*)^−1^. The hatched grey area refers to the discussion of this picture in comparison to Figure 4 in the text.

But how can an initial predominant *R*-selectivity be shifted to an *S*-selectivity, as was observed in the case of the *Ll*KdcA-catalyzed formation of acetoin? To answer this question, the stereoselectivity of the (*S*)-HPP production in *Ll*KdcA has to be considered. Here the acetaldehyde also has to be arranged in an antiparallel manner to the donor aldehyde. The *S*-pocket in *Ll*KdcA is blocked by amino acid side chains and thus not accessible according to the available crystal structure.^12^ However, also in a buffered system the enzyme is able to produce small amounts of the *S*-product, yielding an *ee* of 95.6% for (*R*)-HPP (Figure 4, Figure 8). This means that 97.8% of the *R*-product and 2.2% of the *S*-product are synthesized. Maybe an antiparallel orientation of the acetaldehyde is possible without a distinct *S*-pocket because of the small size of the methyl group. These data might, on the other hand, also suggest that *Ll*KdcA possibly offer something like an alternative *S*-pathway. This alternative *S*-pathway has been discussed recently for the *Ap*PDC.11d Here, we would expect not an antiparallel *Re*-site attack to yield the *S*-product, but a parallel *Si*-attack from the rear of the ThDP as shown in Figure 7.

The other explanation could be that it is not *S*-selectivity that is enhanced by the organic solvent, but the *R*-pathway (Figure 2, A) that is blocked, which reduces the *R*-product and therefore enhances the *ee* of the *S*-product. An indication of this is the fact that EtOH (20 vol%) and THF (2.5 vol%) not only increase the *S*-selective production of acetoin (5.9% *ee* and unselective, respectively), but simultaneously reduce the overall activity of *Ll*KdcA for acetoin (Supporting InformationSupporting Information, Figure S2). This explanation also holds for effects observed with *Pp*BFDH281A: 7 vol% EtOAc shifts the initial *ee* from 47% (*S*)-HPP to 17% (*S*)-HPP. This observation can be explained by a partial suppression of the *S*-path, which is accompanied by a reduced overall HPP-forming activity from 1.85 mM in the buffer control to 0.45 mM in the presence of 7 vol% EtOAc.

Although the solvent-induced shifts in stereoselectivity seem to be pronounced (Figure 4), analyses of the free energy of activation yield a different picture, since the energy differences in the moderate range of the *ee* are much lower compared to those above 90% *ee* (for the logarithmic correlation of the free energy of activation and *ee* see Supporting Information, Figure S8). Figure 8 shows the *ee* of the different products correlated with the respective ΔΔG values.

Although for the initial *ee* values in the buffer controls in the range of 50% (*S*) to 30% (*R*) (Figure 4, hatched gray area on the x-axis) significant *ee* shifts were observed, they correlate with only very small changes of the free energy of activation (Figure 8, hatched gray area on the x-axis). The situation is different with high *ee* values (>90%), where the energy required for increasing the *ee* value further is much higher. This is due to the fact that ΔG increases logarithmically with increasing *ee*s (Supporting Information, Figure S8).

A comparison of Figure 4 and Figure 8 demonstrates that organic solvents are able to induce a pronounced change in stereoselectivity in almost all tested enzymatic reactions. The energy difference induced by a change of the reaction medium is high enough to change a low *ee* to the opposite enantiomer [e.g., *Ll*KdcA in buffer 19.7% (*R*)-acetoin to 21% (*S*)-acetoin in 30 vol% acetone] as well as a high *ee* into an even higher one [e.g., *Pf*BAL in buffer: 99.8 (*R*)-HPP to 99.9 (*R*)-HPP in 30 vol% acetone]. However, the energy differences are not sufficient to decrease the stereoselectivity in the case of very high *ee* values (>99.9%). To obtain an idea of how strong this energy is: a hydrogen bond has a strength of −8.6 kcal mol^−1^.^28^ The strongest impact found in the present study was 1/2 of this strength (*Pp*BFDH281A enhancement of the *ee* for PAC).

In Figure 4, the value, above which no influence on the *ee* was observed, seemed to be >95% *ee*, but in this plot it now becomes obvious that this value was misleading since higher energy has to be expended to increase the already high *ee* values even further.

It seems that a complex interaction between several structural and kinetic factors shifts the chemo- and stereoselectivity. Our results show that medium engineering has a high impact on the selectivity of ThDP-dependent enzymes as well as on the final product concentration and is therefore a good tool for complementing enzyme and reaction engineering in biotransformations to improve or vary an enzyme for a certain reaction. It further demonstrates that the use of organic cosolvents should be carefully planned, as the solvents may compete with the substrate(s) for binding sites or show further influences in the enzyme active site.

In the case of the ThDP-dependent enzymes studied, the improvement of the stereoselectivity is not only observed with one solvent. Often we saw an improvement of the *ee* in the presence of EtOH, acetone, DMSO and *i*Prop. With acetone, for example, we found an inversion from (*R*)-acetoin to (*S*)-acetoin by *Ll*KdcA catalysis. The same solvent was also able to increase an almost perfect (*R*)-HPP *ee* in *Pf*BAL catalysis from 99.8% to 99.9% *ee*.

The solvents that never increased any of the productivities of ThDP-dependent enzymes significantly were: THF, MTBE, MIBK and DIPE. For some reactions these solvents were inert and could be used to merely improve the solubility of aromatic compounds. Due to the limited solubility of these solvents in buffer this effect is not very pronounced. All other cosolvents yielded either an increase or a decrease in the final product concentration. Here, a test for the desired application is more meaningful. However, in order to improve the overall activity of a ThDP-dependent enzyme, priority should be given to testing DMSO. For five reactions, we found an improvement in performance (*Ll*KdcA: acetoin from 2.3 mM in buffer to 25.4 mM, *Ap*PDCE469G: acetoin from 0.7 mM to 1.1 mM, *Pp*BFDH281A: benzoin from 2.5 mM to 3.4 mM, *Pf*BAL: HPP from 3.6 mM to 8.1 mM, *Ap*PDC: PAC from 1.4 mM to 2.6 mM) in mixed carboligation reactions.

## Conclusions

Taking together, the chemical nature of the organic solvent and its concentration has a crucial impact on the enzymatic reaction. Our data show that this effect cannot fully be generalized although we work within an enzyme family with very similar structures and mechanisms. An organic cosolvent as solubility enhancer for hardly soluble compounds should not be used, until the influence of this cosolvent on the enzyme behaviour has been examined.

### Outlook

To verify the hypothesis that the organic solvents bind specifically to the active site and especially to the *S*-pocket of the ThDP-dependent enzyme *Ap*PDCE469G, molecular modelling studies are currently being conducted. In this way, we try to identify potential binding sites for the solvents in the active site or on the surface of the enzyme. In addition, the flexibility of the ThDP-dependent enzymes in water-miscible organic solvents will be studied further.

## Experimental Section

### Enzymes

All enzymes were purified by Ni-chelate chromatography as described elsewhere (*Pf*BAL,13b *Pp*BFD,2d *Pp*BFDH281A: purified as *Pp*BFD, *Ll*KdcA,^12^ *Ap*PDC/*Ap*PDCE469G11d) and used as lyophilizates.

### Reactions

All reactions were performed at an 800-μL scale. The substrates benzaldehyde (18 mM final concentration) and acetaldehyde [18 mM (*Ap*PDC, *Ap*PDCE469G, *Pf*BAL)/180 mM (*Ll*KdcA, *Pp*BFD, *Pp*BFDH281A) final concentration] were dissolved in triethanolamine buffer [50 mM, 0.1 mM ThDP, 2.5 mM MgSO_4_, pH 7.5 (for all enzymes except *Pf*BAL) or pH 8.0 (for *Pf*BAL)]. 0.02 mg/mL (*Pf*BAL)/0.1 mg/mL (for all other enzymes) was used. If the productivity was so low that the stereoselectivity could not be determined reliably, the approach was repeated with 0.4 mg/mL enzyme. The catalyst was dissolved in buffer and finally added to the reaction mixture. Reactions were performed in a thermomixer (Eppendorff, Germany) for 24 h at 20 °C.

The reactions were performed in 1–4 replicates. The error bars are given in the plots in the Supporting Information.

Reaction batches containing organic cosolvents were prepared as described above, but the volume of the buffer was reduced according to the volume of the respective solvent.

### Sample Preparation and Analysis

***Achiral HPLC:*** 200 μL of the sample were mixed with an equal amount of acetonitrile (v/v) containing 2-methoxybenzaldehyde as internal injection standard. Achiral HPLC analysis was performed with a Dionex P680 HPLC pump connected to an ASI-100 automated sample injector and a Dionex UVD170U detector. A LiChrosphere 100 RP-8 (5 μm) Hibar 250–4 (Merck KGaA, Germany) column was operated with a flow of 1 mL min^−1^, an injection volume of 20 μL and a temperature of 25 °C. The mobile phase was a mixture of 25–60% acetonitrile (ACN) and water (min 0–12: 25% ACN, min 12–13: gradient 25%–60% ACN, min 13–20: 60% ACN, min 20–23: gradient 60%–25% ACN, min 23–25: 25% ACN). The products of the reaction mixture were detected at 200 nm (PAC) and 250 nm (HPP, benzoin, benzaldehyde) with the following typical retention times: PAC: 11.2 min, HPP: 13.6 min, benzaldehyde: 21 min, benzoin: 23.6 min. (For the detection of acetoin chiral GC was applied). The system was calibrated by at least three independent series of measurements until the standard deviation of the calibrations to each other was smaller than 10%. Benzaldehyde and benzoin are commercially available (Sigma Aldrich, Steinheim, Germany), while PAC and HPP were synthesized as described elsewhere.^29^

***Chiral HPLC:*** 200 μL of the sample were mixed with an equal amount of an *n*-heptane:*i*Prop [75:25 (v/v)] mixture. After extraction 20 μL of the organic phase was injected. Chiral analysis was performed on an HPLC system with a Gynkotek high-precision pump model 480, a Dionex GINA 50 autosampler, a Dionex UVD170U detector and a Gynkotek Degasys DG 1310. The column Chiralpak IC, 4.6 mm×250 mmL, 5 μm (Daicel Chemical IND., LTD, France) was operated with a flow of 0.5 mL min^−1^ using an *i*Prop:*n*-heptane mixture (25:75) at 25 °C. The compounds were detected at a wavelength of 210 nm (PAC) and 250 nm (HPP, benzoin, benzaldehyde) with the following typical retention times: benzaldehyde: 11.3 min, PAC: 12.5 (*R*) 13.1 (*S*), HPP: 14.2 (*R*) 15.6 (*S*), BZ: 16.8 (*R*) 17.8 (*S*).

***Chiral gas GC:*** For the determination of acetoin, 200 μL of the sample were mixed with an equal amount of EtOAc, including the injection standard decane and centrifuged. 1 μL of the organic phase was injected. Acetoin production was monitored on an Agilent Technologies 6890N Network GC system equipped with an F*S*-Cyclodex beta-I/P column. Heating of the oven: 75 °C (5.3 min), 10 °C min^−1^ to 180 °C (maintained for 3 min). Typical retention times: acetoin: 5.3 min (*R*) and 5.6 min (*S*). The analytical systems were calibrated with three independent samples of racemic acetoin (Sigma Aldrich, Steinheim, Germany). The standard deviation of the different calibrations to each other was 4.6%.

### Organic Solvents

The organic solvents used including the applied concentration range, their log P values and solvent excluded volume are listed in Table 1.

## Abbreviations

*Ap*PDC: pyruvate decarboxylase from *Acetobacter pasteurianus*
DCM: dichloromethane
DIPE: diisopropyl ether
DMSO: dimethyl sulfoxide
*ee*: enantiomeric excess
EtOAc: ethyl acetate
EtOH: ethanol
GC: gas chromatography
HPLC: high-performance liquid chromatography
HPP: 2-hydroxy-1-phenylpropan-1-one
*i*Prop: isopropyl alcohol
*Ll*KdcA: branched-chain keto acid decarboxylase from *Lactococcus lactis*
MIBK: methyl isobutyl ketone
MTBE: methyl *tert*-butyl ether
MTHF: 2-methyltetrahydrofuran
PAC: 1-hydroxy-1-phenylpropan-2-one
*Pf*BAL: benzaldehyde lyase from *Pseudomonas fluorescens*
*Pp*BFD: benzoylformate decarboxylase from *Pseudomonas putida*
rpd: relative product distribution
TCM: trichloromethane
THF: tetrahydrofuran

## Supplementary material

Detailed facts of importance to specialist readers are published as ”Supporting Information”. Such documents are peer-reviewed, but not copy-edited or typeset. They are made available as submitted by the authors.
