# Supplementary material for: Influence of Organic Solvents on Enzymatic Asymmetric Carboligations
Source: Adv Synth Catal. 2012 Oct 4;354(14-15):2805–20. doi: 10.1002/adsc.201200284 (PMC3549479; doi:10.1002/adsc.201200284)
Supplement: Supplementary file 1 [file adsc0354-2805-SD1.pdf]

Advanced Synthesis & Catalysis

Supporting Information

© Copyright Wiley-VCH Verlag GmbH & Co. KGaA, 69451 Weinheim, 2012

Influence of organic solvents on enzymatic asymmetric carboligations

Tina Gerhards,[a] Ursula Mackfeld,[a] Marco Bocola,[b] Eric von Lieres,[a] Wolfgang Wiechert,[a] Martina Pohl,[a] Dörte Rother [a,*]

[a] Institute of Bio- and Geosciences, IBG-1: Biotechnology, Forschungszentrum Jülich GmbH, 52425 Jülich, Germany
Fax: +49-2461-613870; phone: +49-2461-616772; e-mail: do.rother@fz-juelich.de
[b] Institute of Biotechnology, RWTH Aachen University, Worringerweg 1, 52074 Aachen, Germany

Supporting Information

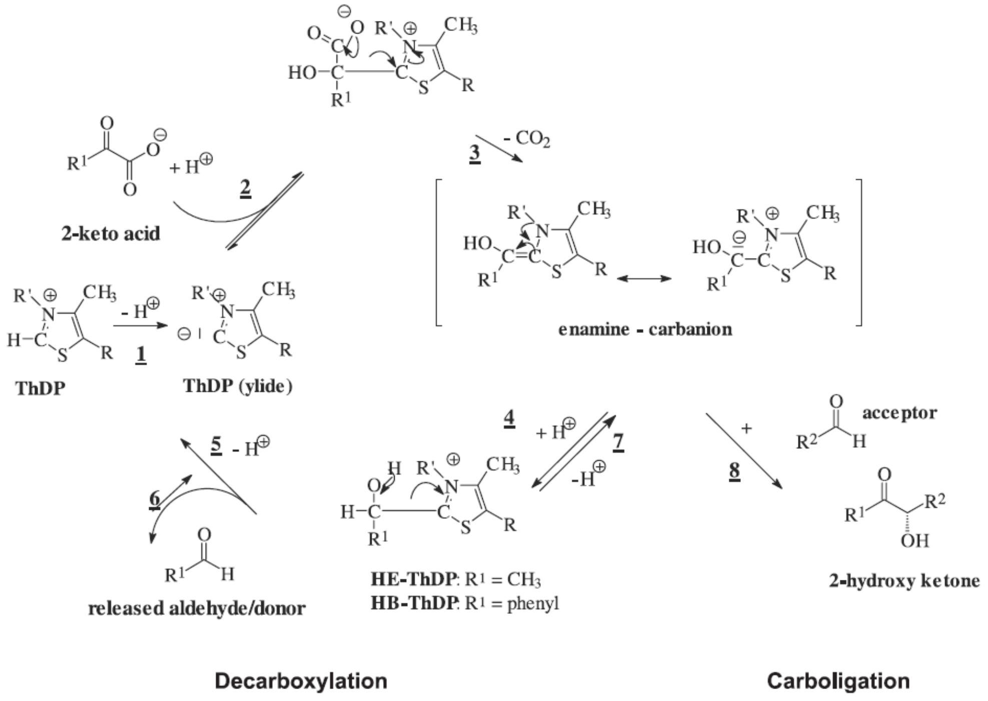

Figure S 1: Reaction mechanism of BFD and PDC[25]. The reaction cycle is started by deprotonation of ThDP bound to the enzyme (1). The ylide is able to bind a 2-keto acid (pyruvate, R1 = methyl; benzoylformate, R1 = phenyl) via nucleophilic addition to the carbonyl group (2). The resulting adduct decarboxylates to an enamine carbanion (3). Protonation of this species yields hydroxyethyl-(HE)-ThDP or hydroxybenzyl-(HB)-ThDP (4), which subsequently eliminates the corresponding aldehyde (PDC, acetaldehyde; BFD, benzaldehyde) upon protonation and regenerates the ThDP ylide (5). Decarboxylation and carboligation are assumed to have a common intermediate, the enamine carbanion. In addition to the decarboxylation route (2 and 3), this species can be generated by direct addition of a (donor) aldehyde to the ThDP ylide (route 6 and 7). Addition of a further (acceptor) aldehyde (8) leads to the formation of 2-hydroxy ketones

How to read Figure 3B?

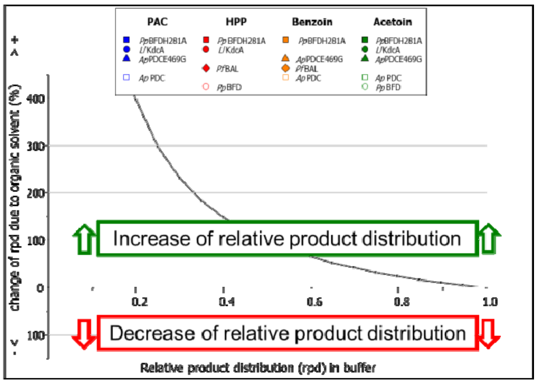

X-axis: relative product distribution ([A]/([A]+[B]+[C]+[D])) in buffer control without addition of any organic solvent.
Y-axis: change of relative product distribution (%) due to the presence of an organic solvent
Each color refers to one of the possible products, each symbol to one of the six enzymes
Y > 0 : increase of relative product distribution, Y > 0 : decrease of relative product distribution
Due to the percentage plotting, the black line in the upper part of the plot cannot be overstepped

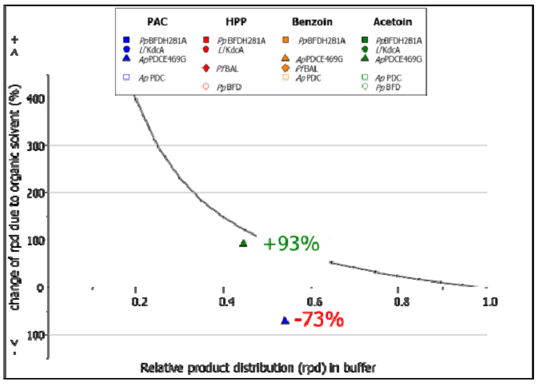

Example: *Ap*PDCE469G in buffer: rpd (PAC) = 0.56; prd (acetoin) = 0.44
Induced change (%) due to EtOH: PAC = -73%; acetoin = 93 %
PAC: starting point on x-axis: 0.56, decrease of 73 % (plot, blue triangle)
Acetoin: starting point on x-axis: 0.44, increase of 93 % (plot, green triangle)

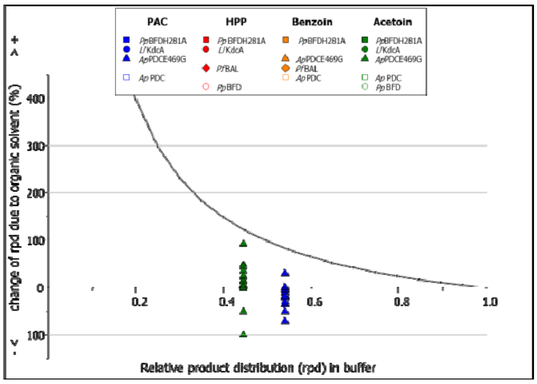

Whole data set for *Ap*PDCE469G including PAC and Acetoin

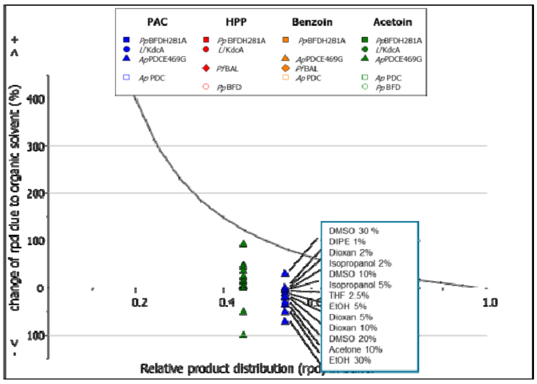

Every data point represents the influence of one organic solvent in one concentration

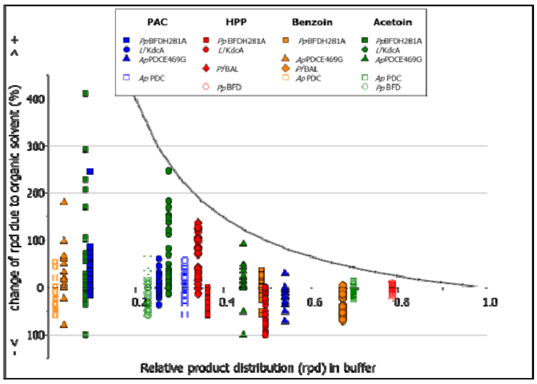

All enzymes with all their products influenced by all organic solvents

Raw data of all the chemo- and stereoselectivity measurements are provided in this supporting information. Data are ordered according to the different enzymes.

In the upper part of the diagram, the results for chemoselectivity are given. On the x-axis the different solvents are sorted according to the respective concentration (v/v) (buffer: buffer control without any organic solvent, we: reaction mixtures without enzyme, ws: reaction mixtures without substrates). On the y-axis, the concentration of every product after 24 h is given in mM.

Results for stereoselectivity are placed below. The x-axis again shows the different solvents in different concentrations and the enantiomeric excess (*ee*) is plotted on the y-axis. Negative values correspond to an excess of the *S*-enantiomer.

In some cases, the data collection was split. Independent controls were carried out for every time point. In the graphs, only one sample without enzyme (we) and without solvent (ws) is shown because these samples never showed activity. However, the buffer controls (buffer) without solvent addition did differ. Therefore every result was analyzed in relation to its own buffer control. In the plots, the buffer controls for the different measurements can always be found in a row at the end of each split set.

AC	acetoin
Acet	acetone
*Ap*PDC	pyruvate decarboxylase from *Acetobacter pasteurianus*
buffer	buffer control without any additives
BZ	benzoin
DCM	dichloromethane
Diox	dioxane
DIPE	diisopropylether
DMSO	dimethyl sulfoxide
ee	enantiomeric excess
EtOAc	ethyl acetate
EtOH	ethanol
HPP	2-hydroxy-1-phenyl-propan-1-one
Isop	isopropanol
*Ll*KdcA	branched-chain keto acid decarboxylase from *Lactococcus lactis*
MIBK	methyl isobutyl ketone
MTBE	methyl *tert*-butyl ether
MTHF	2-methyltetrahydrofuran
PAC	1-hydroxy-1-phenyl-propan-2-one
*Pf*BAL	benzaldehyde lyase from *Pseudomonas fluorescens*
*Pp*BFD	benzoylformate decarboxylase from *Pseudomonas putida*
TCM	trichloromethane
THF	tetrahydrofuran
we	without enzyme
ws	without solvent

*Ll*KdcA

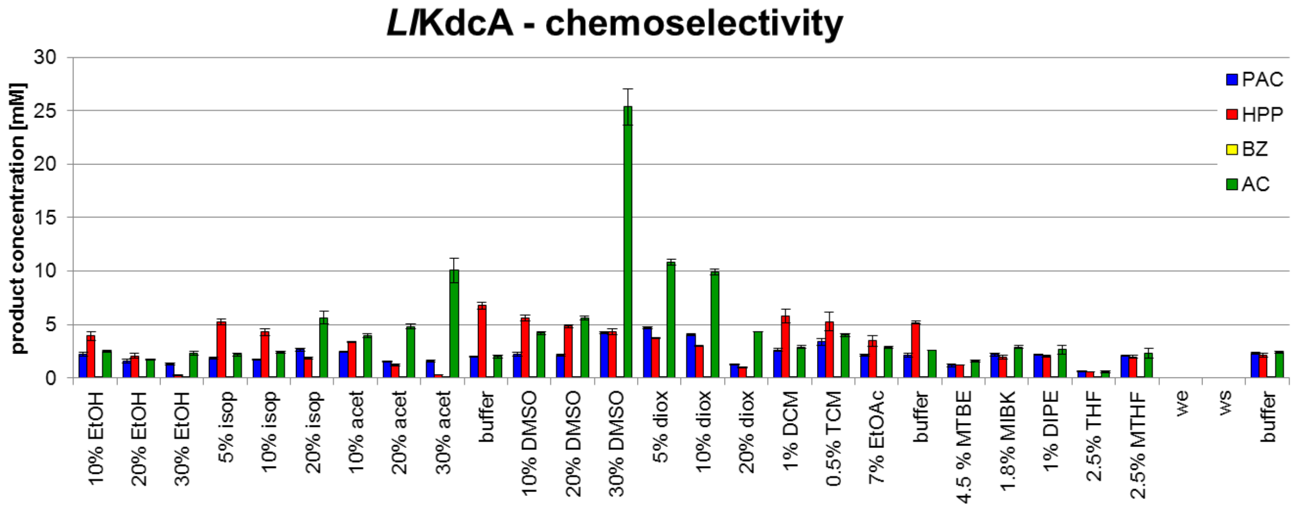

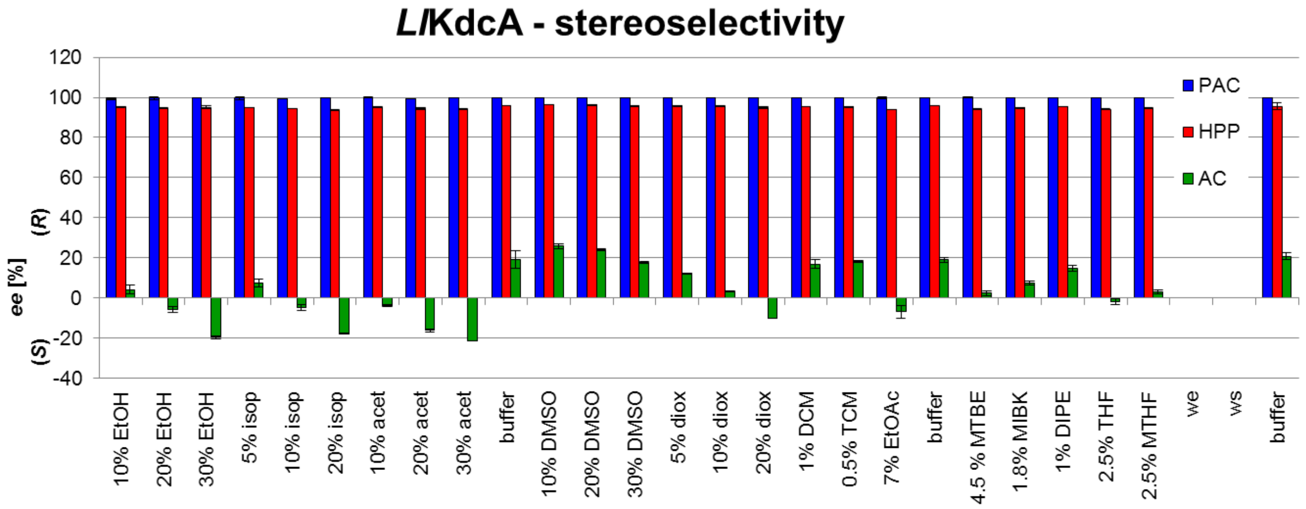

Figure S 2: *Ll*KdcA: chemo- and stereoselectivity. Reaction conditions: TEA buffer pH 7.5, 0.1/ 0.4 mg/mL enzyme, 180 mM acetaldehyde, 18 mM benzaldehyde, measured as triplicates. Negative values in stereoselectivity refer to an excess of the *S*-enantiomer.

*Pp*BFD

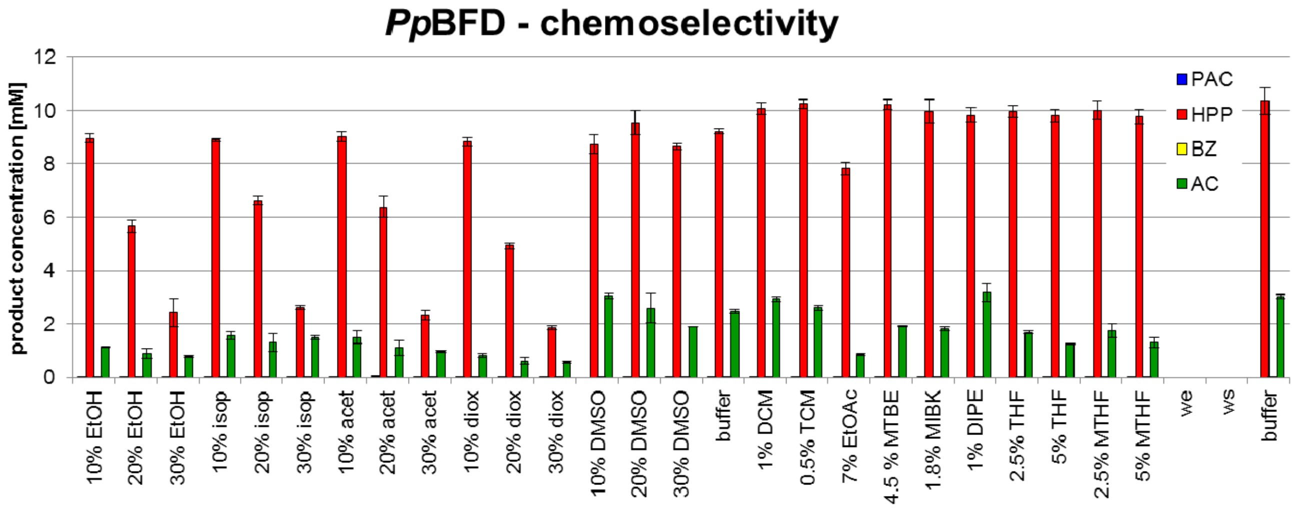

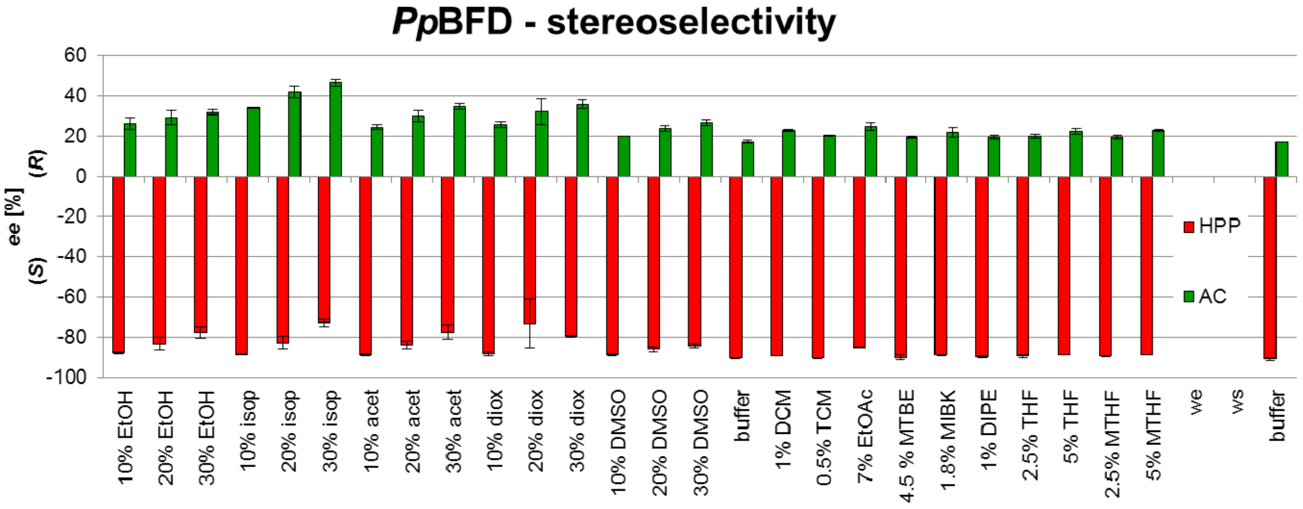

Figure S 3: *Pp*BFD: chemo- and stereoselectivity. Reaction conditions: TEA buffer pH 7.5, 0.1 mg/mL enzyme, 180 mM acetaldehyde, 18 mM benzaldehyde, measured as triplicates. Negative values in stereoselectivity refer to an excess of the *S*-enantiomer.

*Pp*BFDH281A

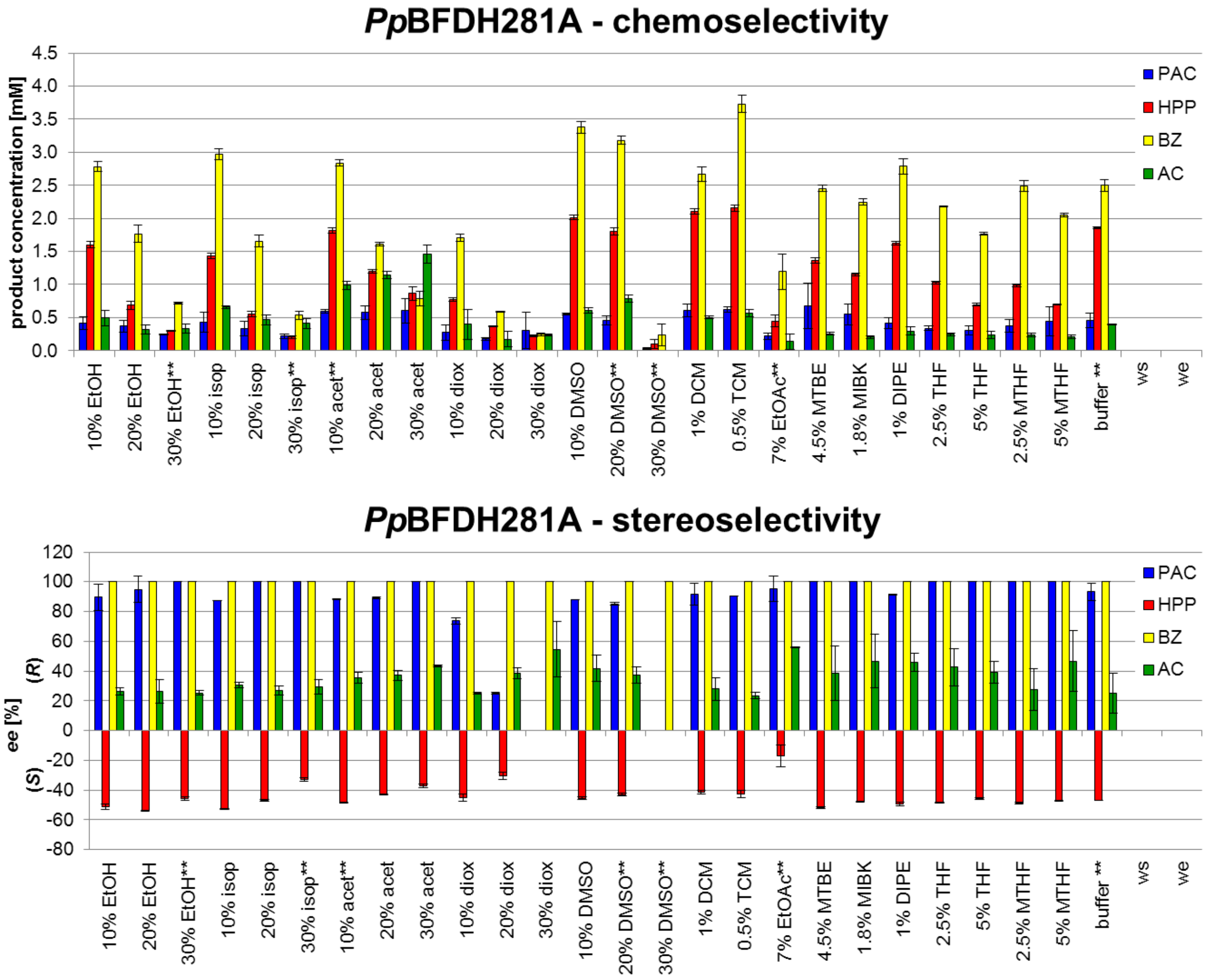

Figure S 4: *Pp*BFDH281A: chemo- and stereoselectivity. Reaction conditions: TEA buffer pH 7.5, 0.1 mg/mL enzyme, 180 mM acetaldehyde, 18 mM benzaldehyde, measured as triplicates, except for **: here, the PAC result derives from 2 measurements (only for chemoselectivity). Negative values in stereoselectivity refer to an excess of the *S*-enantiomer.

*Ap*PDC

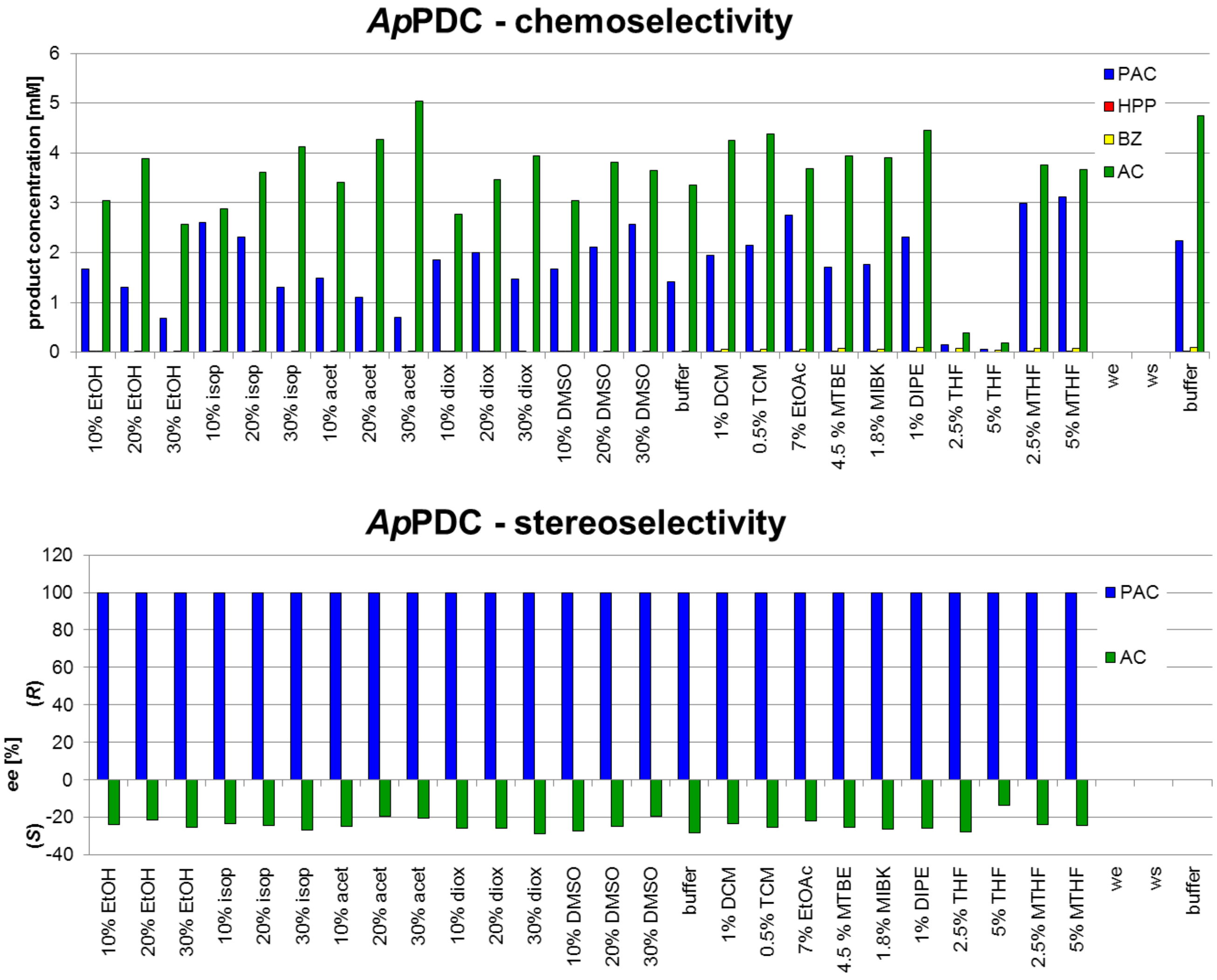

Figure S 5: *Ap*PDC: chemo- and stereoselectivity. Reaction conditions: TEA buffer pH 7.5, 0.1 mg/mL enzyme, 18 mM acetaldehyde, 18 mM benzaldehyde. Negative values in stereoselectivity refer to an excess of the *S*-enantiomer.

*Ap*PDCE469G

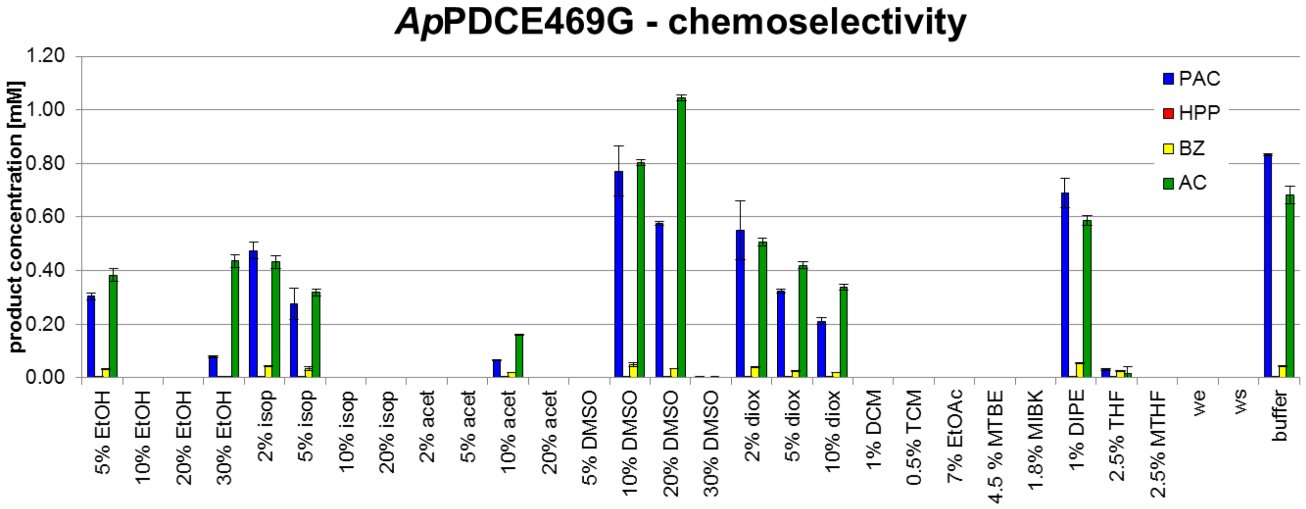

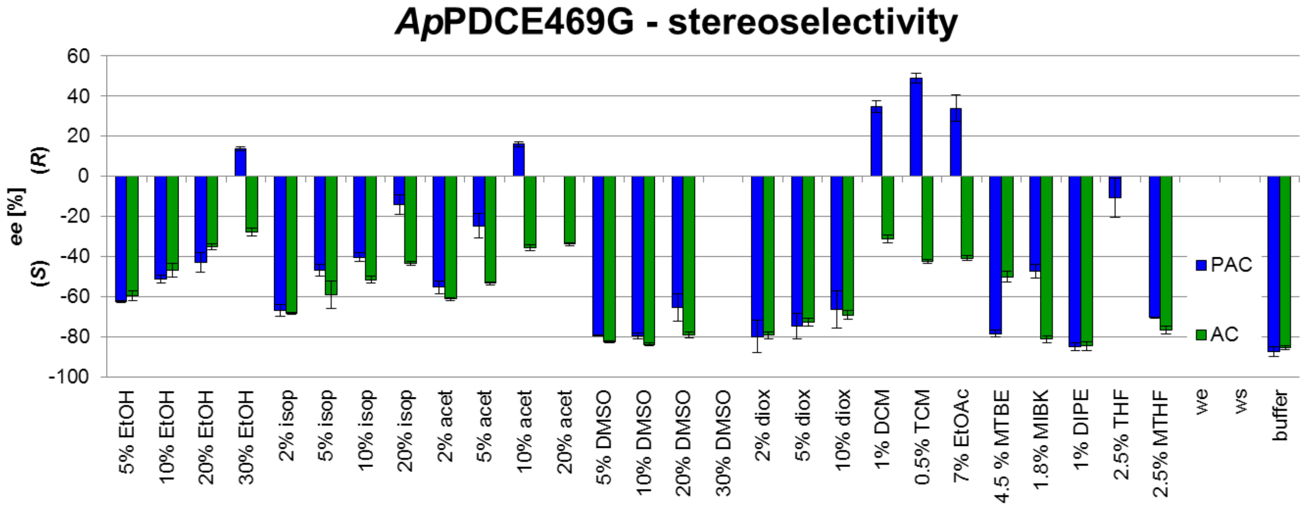

Figure S 6: *Ap*PDCE469G: chemo- and stereoselectivity. Reaction conditions: TEA buffer pH 7.5, 0.1/ 0.4 mg/mL enzyme, 18 mM acetaldehyde, 18 mM benzaldehyde, measured as triplicates. Negative values in stereoselectivity refer to an excess of the *S*-enantiomer.

*Pf*BAL

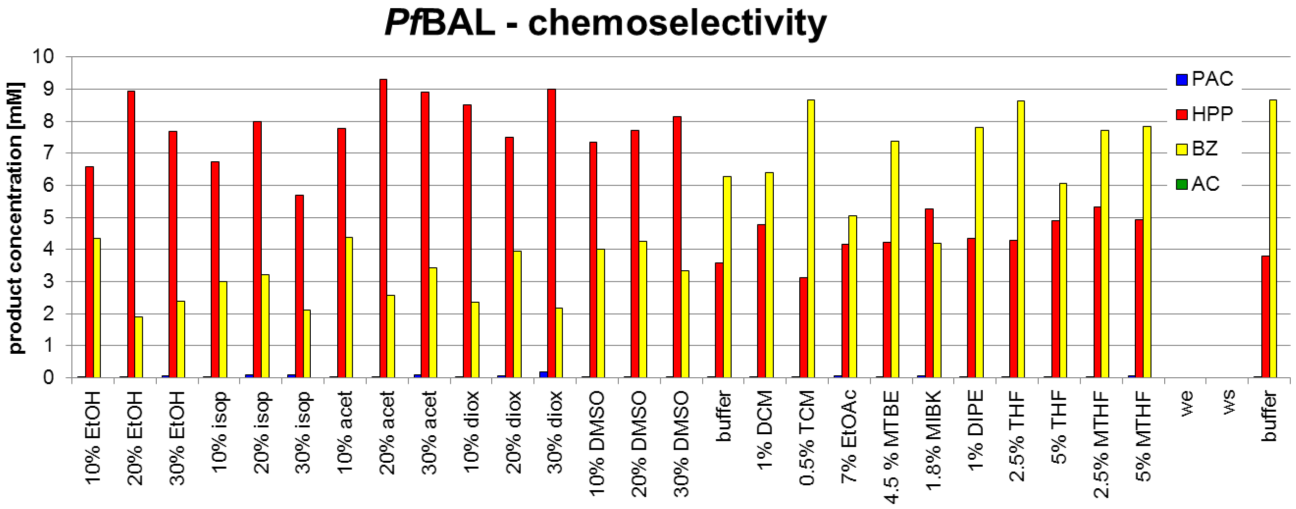

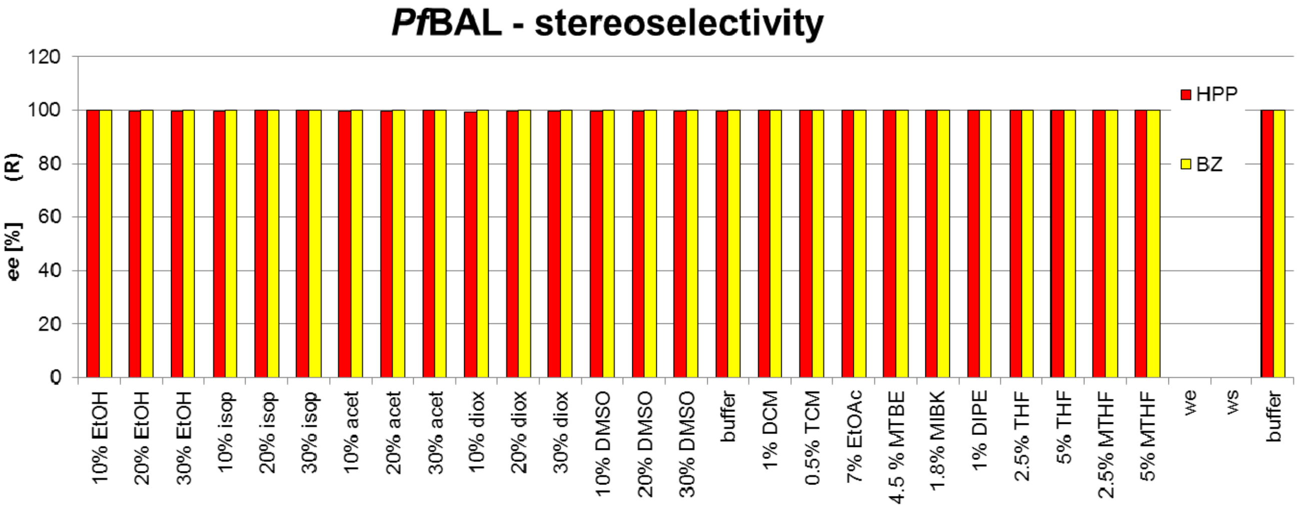

Figure S 7: *Pf*BAL: chemo- and stereoselectivity. Reaction conditions: TEA buffer pH 8.0, 0.02 mg/mL enzyme, 18 mM acetaldehyde, 18 mM benzaldehyde. Negative values in stereoselectivity refer to an excess of the *S*-enantiomer.

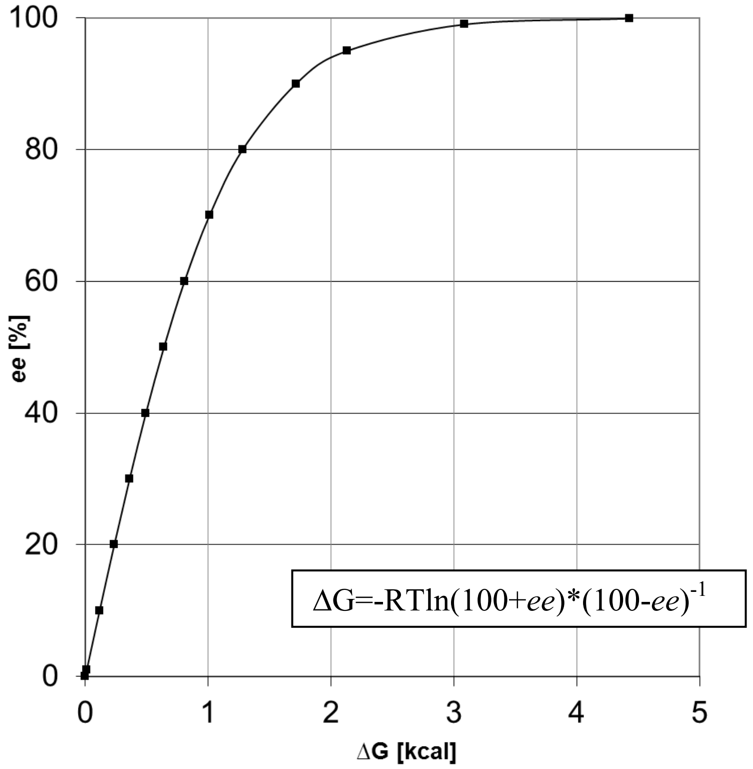

Figure S 8: ΔG values of different enantiomeric excesses (*ee*s).

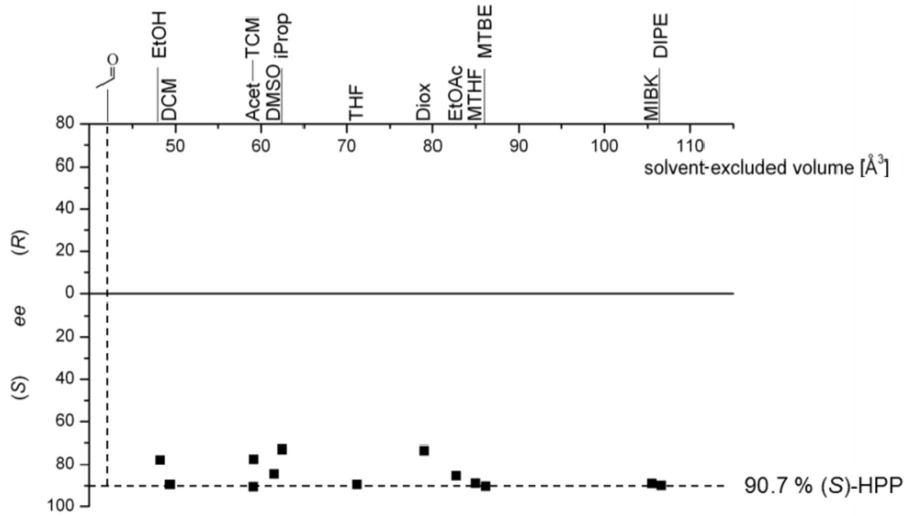

Figure S 9: Influence of different organic cosolvents (always in the highest used concentration which yielded reliable stereoselectivity values sorted according to their size-excluded volume [Å^3]) on the stereoselectivity of the PAC formation catalyzed by *Pp*BFD. In buffer, (*S)*-HPP is obtained with an *ee* of 90% (horizontal dashed line, compare Figure 3). Also the size of the acetaldehyde is given (vertical dashed line), because its side chain has to enter and fit into the *S*-pocket to yield (*S*)-HPP.

Table S 1 Organic solvents and concentrations in vol% and mol/L.

Organic solvent	Density	Mw (g/mol)	Concentration (vol%)	Concentration (mol/L)
EtOH	0.79	46.07	5	1.37
			10	2.75
			20	5.50
			30	8.24
DCM	1.33	84.93	1	0.09
Acetone	0.79	58.08	2	0.44
	0.79	58.08	5	1.09
	0.79	58.08	10	2.18
	0.79	58.08	20	4.36
	0.79	58.08	30	6.54
TCM	1.48	119.38	0.5	0.03
DMSO	1.1	78.13	5	0.58
	1.1	78.13	10	1.16
	1.1	78.13	20	2.33
	1.1	78.13	30	3.49
*i*Prop	0.78	60.1	5	1.07
	0.78	60.1	10	2.13
	0.78	60.1	20	4.27
	0.78	60.1	30	6.40
THF	0.89	72.11	2.5	0.39
	0.89	72.11	5	0.78
dioxane	1.03	88.11	5	0.55
	1.03	88.11	10	1.10
	1.03	88.11	20	2.20
	1.03	88.11	30	3.31
EtOAc	0.894	88.11	7	0.89
MTHF	0.854	86.13	2.5	0.34
	0.84	86.13	5	0.68
MTBE	0.74	88.15	4.5	0.69
MIBK	0.8	100.16	1.8	0.22
DIPE	0.72	102.18	1	0.14
